# Genetic mapping and legume synteny of aphid resistance in African cowpea (*Vigna unguiculata* L. Walp.) grown in California

**DOI:** 10.1007/s11032-015-0254-0

**Published:** 2015-01-21

**Authors:** Bao-Lam Huynh, Jeffrey D. Ehlers, Arsenio Ndeve, Steve Wanamaker, Mitchell R. Lucas, Timothy J. Close, Philip A. Roberts

**Affiliations:** 1 Department of Nematology, University of California, Riverside, CA 92521 USA; 2 Present Address: Bill and Melinda Gates Foundation, Seattle, WA 98102 USA; 3 Department of Botany and Plant Sciences, University of California, Riverside, CA 92521 USA

**Keywords:** Biotic stress, Legume, Cowpea aphid, Physical mapping, *Vigna unguiculata*

## Abstract

The cowpea aphid *Aphis craccivora* Koch (CPA) is a destructive insect pest of cowpea, a staple legume crop in Sub-Saharan Africa and other semiarid warm tropics and subtropics. In California, CPA causes damage on all local cultivars from early vegetative to pod development growth stages. Sources of CPA resistance are available in African cowpea germplasm. However, their utilization in breeding is limited by the lack of information on inheritance, genomic location and marker linkage associations of the resistance determinants. In the research reported here, a recombinant inbred line (RIL) population derived from a cross between a susceptible California blackeye cultivar (CB27) and a resistant African breeding line (IT97K-556-6) was genotyped with 1,536 SNP markers. The RILs and parents were phenotyped for CPA resistance using field-based screenings during two main crop seasons in a ‘hotspot’ location for this pest within the primary growing region of the Central Valley of California. One minor and one major quantitative trait locus (QTL) were consistently mapped on linkage groups 1 and 7, respectively, both with favorable alleles contributed from IT97K-556-6. The major QTL appeared dominant based on a validation test in a related F2 population. SNP markers flanking each QTL were positioned in physical contigs carrying genes involved in plant defense based on synteny with related legumes. These markers could be used to introgress resistance alleles from IT97K-556-6 into susceptible local blackeye varieties by backcrossing.

**Electronic supplementary material:**

The online version of this article (doi:10.1007/s11032-015-0254-0) contains supplementary material, which is available to authorized users.

## Introduction

Cowpea aphid (CPA, *Aphis craccivora* Koch) is a major sap-sucking insect pest of cowpea (*Vigna unguiculata* L. Walp.), an important food, fodder and cover crop grown in Sub-Saharan Africa and other warm-to-hot regions worldwide (Ehlers and Hall 1997; Hall et al. 2003). CPA inflicts damage by direct feeding and injecting toxic saliva into phloem, leading to stunted growth or death of the plant. At high infestation levels, honeydew released by CPA can block plant respiration and stimulate development of black mold, thereby reducing photosynthesis. CPA is also responsible for spreading viral diseases such as cowpea aphid-borne mosaic virus (Atiri et al. 1986). Biological control alone is not adequate because natural enemies often appear when CPA infestation is already high and causing serious damage. Applying pesticides early in the season prevents CPA infestation and colonization but beneficial insects can be destroyed, leading to outbreaks of other insect pests. In fact, pesticide application is not a common practice in low-input farming systems in Africa (Souleymane et al. 2013). Improving cultivars by adding in resistance through breeding promises a sustainable strategy for aphid control not only in cowpea but also in many other crop species (Huynh et al. 2013; Smith and Chuang 2014).

In the USA, blackeye-type dry-grain cowpea is grown in the Central Valley of California where CPA is prevalent, in part due to the large-scale production of alfalfa, a favorite host of CPA. All current California blackeye cultivars are susceptible to CPA and require pesticide treatments during early vegetative and flowering stages. Breeding resistant blackeye cultivars must rely on African cowpea resistance donors (Hall et al. 2003) and can take advantage of new knowledge of trait inheritance. In earlier studies, Pathak (1988) and Githiri et al. (1996) reported that there were two independent genes controlling CPA resistance in African cowpea based on quantitative analyses of segregating populations derived from different combinations of resistant and susceptible parents. Using restriction fragment length polymorphism (RFLP) mapping, Myers et al. (1996) identified RFLP markers with major effects on CPA resistance using an F2 population derived from a susceptible parent and the resistant cultivar IT84S-2246-4. However, there has been no further report on implementation of these RFLP-linked traits in cowpea breeding, and there have been observations in West Africa of the breakdown of resistance in IT84S-2246-4 (Fatokun, pers. comm.). Another consideration is that those earlier genetic studies were performed in greenhouses using specific locally collected aphid colonies whose biotype status is not known and may not be generally representative of CPA populations in cowpea fields in Africa and the USA. Indeed, up to 97 single-nucleotide polymorphisms (SNPs) were detected among CPA populations collected at 15 locations in West Africa (Agunbiade et al. 2013).

Cowpea aphid also feeds on a range of other legumes, such as *Medicago truncatula*, alfalfa, chickpea, lentil, lupin, peanut and many pasture legume species (Edwards 2001; Nair et al. 2003). However, reports on genetic control of CPA resistance in these hosts are rare. To date, CPA-resistance sources and major quantitative trait loci (QTL) have been reported only for peanut (Herselman et al. 2004) and *M. truncatula* (Kamphuis et al. 2012). Genetic mapping for CPA resistance in cowpea would help identify syntenic regions in other legumes, as they may confer similar physiological responses to CPA infestation (Kamphuis et al. 2013).

In this study, we aimed to identify QTL for CPA resistance in cowpea using field data collected from genetic materials grown under aphid-unprotected conditions in the Central Valley of California over two years. Gene-associated SNP markers (Muchero et al. 2009) were used in both genetic and physical mapping of the QTL followed by syntenic analysis with other legumes for candidate-gene identification. The findings provide a foundation for gene cloning and marker-assisted backcrossing (MABC) for developing CPA-resistant cowpea cultivars for California and other regions affected by similar CPA biotypes.

## Materials and methods

### Genetic materials and resistance phenotyping

Field-based assays for genetic mapping involved 92 recombinant inbred lines (RILs) (F8) derived from a cross between susceptible California blackeye cultivar ‘California Blackeye 27’ (CB27), which was bred by University of California–Riverside (UCR) (Ehlers et al. 2000), and a resistant breeding line IT97K-556-6 from the International Institute of Tropical Agriculture (IITA) breeding nursery in Nigeria. The RIL population and two parents were planted together under irrigated conditions in the field at the University of California Kearney Agricultural Research and Extension Center (UC-KARE) in Parlier, California, in 2012 and 2013. No pesticides were applied during the course of the experiments. In 2012, the population was planted on May 24 in a randomized complete block design with four blocks. In 2013, the experiment was planted on June 6 in a different field site at UC-KARE but using only one block. In each block, each line was planted in one row of 30 inches in width and 21 feet in length at a density of 3–4 seeds per foot (16 plants/m^2^ on average) using a tractor-mounted planter.

The highly susceptible cowpea cultivar Big Buff was grown throughout the trial sites as aphid spreader rows to attract natural CPA and promote heavy, uniform infestation levels in all plots. Aphid density was estimated based on sampling aphids from plants randomly selected from spreader rows. Canopies of nine plants were cut into a plastic bag and washed in 10 L of deionized water containing 2 mL of liquid detergent. The aphid–water mixture was thoroughly stirred and a sample of 50 mL was filtered on Whatman No.4 filter paper (24 cm diameter). Aphids were counted with the aid of a 10×-illuminated magnifier. The mean of five independent samples was used to determine the average number of aphids per plant or per square meter (16 plants).

Aphid damage symptoms in experimental plots were measured at 50–60 days after planting when aphids infested all spreader rows and caused distinct phenotypic variation among RILs and parents, and again 20 days later after the aphid population diminished and plants started showing recovery. The rating scale was from 0 to 10 based on crown damage and the extent of aphid occurrence applied to more than 50 % of plants in each plot (Additional File 1).

Analysis of Variance (ANOVA) was performed with the software GenStat version 11 (Payne et al. 2008). Factors in the ANOVA model were lines and block, with each of the field locations considered as a block. Broad-sense heritability (trait repeatability) was estimated based on the variance component attributable to variation among lines (*V*_G_) and residual variation (*V*_E_) (*h* ^2^ = *V*_G_/(*V*_G_ + *V*_E_)). Simple linear correlation analysis was used to examine the consistency in damage symptoms between scoring times.

### Linkage analysis and QTL mapping

Marker genotype data for 92 RILs of the CB27 × IT97K-556-6 population were obtained from Lucas et al. (2011) and generated from the Illumina GoldenGate assay of 1,536 genome-wide SNP markers derived from EST sequences (Muchero et al. 2009). Linkage maps were constructed with the software QTL IciMapping 3.1 (http://www.isbreeding.net) using the Kosambi function, RECORD ordering algorithm (Van Os et al. 2005) and alignment with the cowpea consensus genetic map (Lucas et al. 2011) available at HarvEST:Cowpea (http://harvest-web.org/). QTL analysis was also performed with QTL IciMapping using the Inclusive Composite Interval Mapping (ICIM) method (Li et al. 2007; Wang 2009). The ICIM involved three consecutive steps: (1) Single marker analysis was used to select for significant markers (*P* < 0.001) associated with phenotypes, (2) phenotypic values were adjusted for the selected markers except for the two markers flanking the current mapping interval, and (3) the adjusted phenotypic values were used in composite interval mapping which involves testing QTL additive effect and epistatic interaction between QTLs (Yang et al. 2007).

### QTL validation

An F2 population was generated by crossing a blackeye cultivar CB50 (Ehlers et al. 2009), which is highly susceptible to CPA, and a RIL (RIL#41) from the CB27 × IT97K-556-6 population which was homozygous for the favorable (resistance) alleles at both *QAc*-*vu1.1* and *QAc*-*vu7.1*. About 500 F2 seeds were planted at UC-KARE in 2013 on the same field site adjacent to the CB27 × IT97K-556-6 RIL population. The cultivar Big Buff was also planted as spreader rows. A subset of 120 individuals with extreme symptoms, including 80 highly resistant and 40 highly susceptible plants, were genotyped with SNP markers flanking each CPA-resistance QTL using the Kompetitive allele-specific polymerase chain reaction (KASP) assay (LGC Genomics Ltd., Hoddesdon, UK) (Semagn et al. 2014). The marker–phenotype association was visualized with Microsoft Excel.

### Syntenic analysis

Genic sequences harboring cowpea SNP markers were obtained from Muchero et al. (2009) and used as a query to retrieve associated bacterial artificial chromosome (BAC) physical contigs of the cowpea line IT97K-499-35 at HarvEST:Cowpea (http://harvest-web.org/) and cowpea physical mapping database browser (http://phymap.ucdavis.edu/cowpea/). The QTL-bearing BAC sequences were compared using NCBI BLAST+ 2.2.28 with annotated genome sequences of the model legume *M. truncatula* and other legumes (mung bean, common bean, soybean and pigeonpea) which are closely related to cowpea (Choi et al. 2004). Gene models of *M. truncatula* (version Mt4.0v1), soybean (version 275 Wm82.a2.v1) and common bean (version 218) were obtained from Phytozome (http://phytozome.net). Sequences of pigeonpea (draft version) and mung bean (version 6) were accessed at www.icrisat.org/gt-bt/iipg/genomedata.zip and plantgenomics.snu.ac.kr/data/mungbean_data/, respectively.

## Results

### Phenotypic variation in CPA damage

**Fig. 1:**
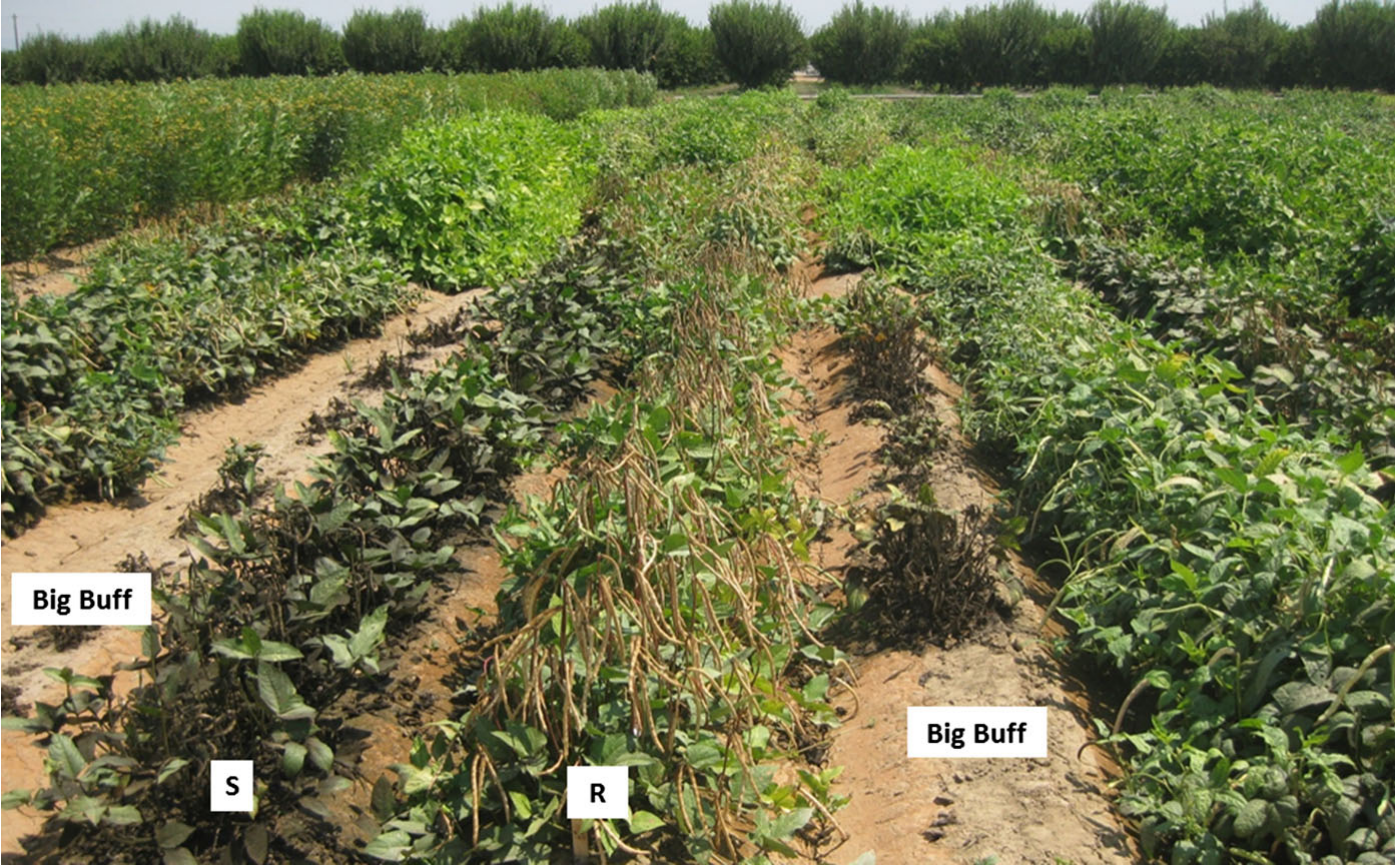
A field-based screening for CPA resistance in the CB27 × IT97K-556-6 RIL population at UC-KARE. Each RIL was planted in a 6-m row. The highly susceptible cv. Big Buff was planted as spreader rows. No pesticide was applied during the course of experiment. Plants shown were at 80 days after planting on May 24, 2012

**Fig. 2:**
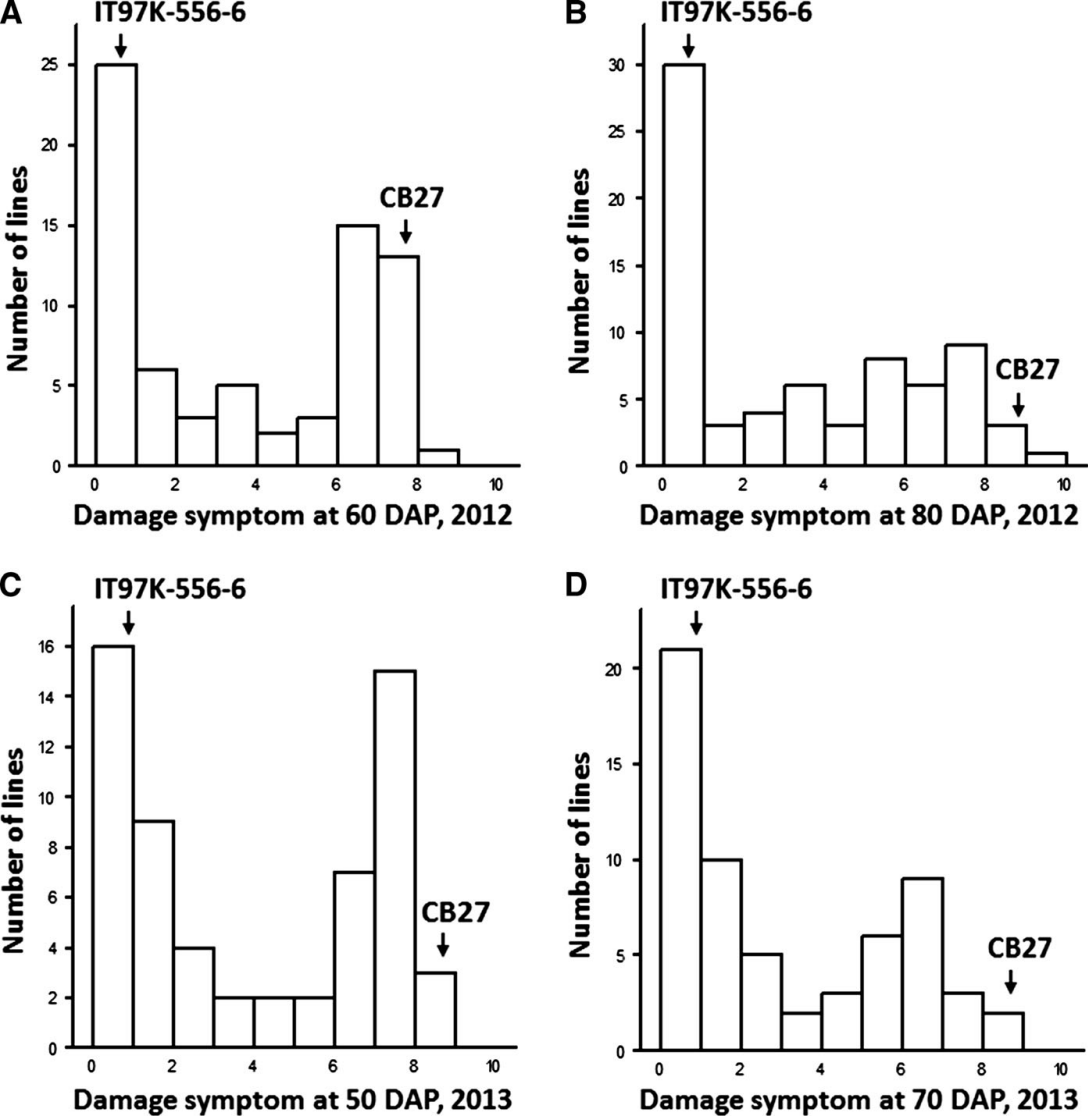
Variation in aphid damage symptoms measured at different days after planting (DAP) among CB27, IT97K-556-6 and their recombinant inbred line population grown at UC-KARE, Parlier, CA, in 2012 and 2013

Aphids began feeding on young cowpea seedlings in the experimental plots about three weeks after planting. Microscopic examination of aphid morphology confirmed that they were typical *A. craccivora* Koch characterized as shiny-black adults and gray nymphs. Severe symptoms of aphid infestation on cowpea plants included dead or stunted plants with black-mold development caused by honeydew excreted from aphids (Additional File 2); the susceptible parent cultivar CB27 was stunted by aphids, while the African breeding line IT97K-556-6 was fully resistant with no to mild symptoms. Damage symptoms of the RILs in field plots were scored at 60 and 50 days after planting (DAP) in 2012 and 2013, respectively. By those dates, CPA had infested all spreader rows and distinct variation in the symptoms was observed among experimental plots. There were approximately 65,000 aphids/plant (including nymphs and adults) in spreader rows (approximately 1,040,000 aphids/m^2^). Damage symptoms were scored again 20 days later when most plants in spreader rows were dead (Fig. 1). There were no symptoms of cowpea aphid-borne mosaic virus during the CPA scoring periods. The phenotypic values of the RIL population were highly consistent among blocks and years (repeatability *h* ^2^ > 0.8) and scoring times (*r* > 0.8, *P* < 0.001). Two major groups of RILs had extreme symptoms (resistant *vs.* susceptible), while other RILs expressed symptoms that were intermediate between the two parents CB27 and IT97K-556-6 (Fig. 2).

### QTLs associated with CPA resistance

**Fig. 3:**
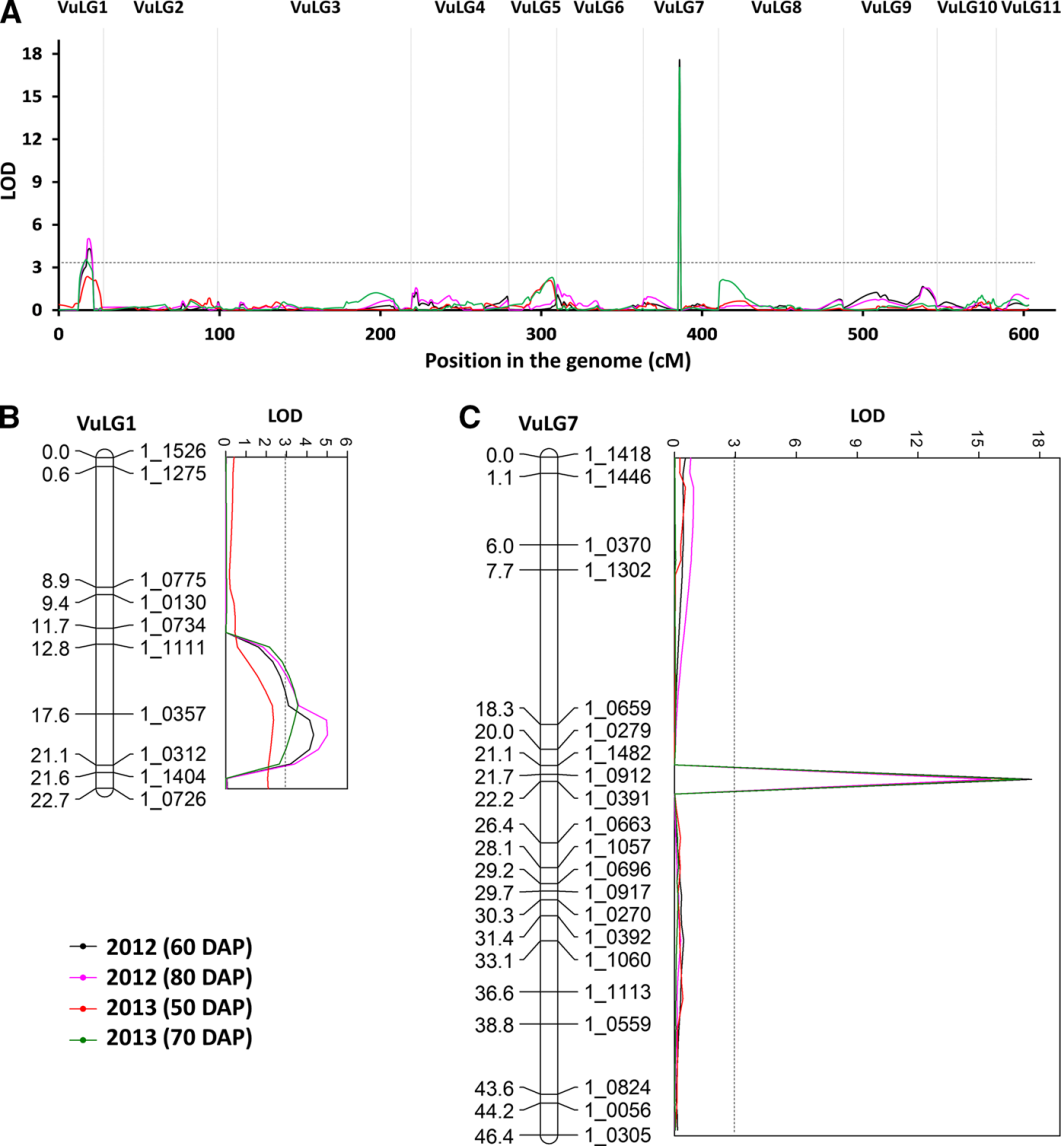
Chromosomal regions associated with aphid damage symptoms measured at different days after planting (DAP) in the CB27 × IT97K-556-6 RIL population grown at UC-KARE, Parlier, CA, in 2012 and 2013: **a** whole genome scan, **b** minor QTL *QAc*-*vu1.1* on LG 1 and **c** major QTL *QAc*-*vu7.1* on LG 7

Two QTLs associated with response to CPA infestation were identified using data recorded at different assay times and growing seasons (Table 1; Fig. 3). The major QTL, *QAc*-*vu7.1,* was located on linkage group 7 of the CB27 × IT97K-556-6 genetic map, explaining approximately 61–66 % of the total phenotypic variation. The minor QTL with a smaller additive effect, *QAc*-*vu1.1,* was located on linkage group 1, explaining approximately 5–13 % of the total phenotypic variation. The linkage group designations (LG 1 and LG 7) on the CB27 × IT97K-556-6 individual map are equivalent to the linkage group designations on the latest version of the cowpea consensus genetic map (Lucas et al. 2011) available at HarvEST:Cowpea (http://harvest-web.org/). Favorable alleles (low-symptom score alleles) were contributed from IT97K-556-6 at both loci. There was no epistatic interaction between two QTLs.

**Table 1:** Chromosomal locations associated with aphid damage symptoms (0–10) measured at different growth stages of the CB27 × IT97K-556-6 RIL population at UC-Kearney Agricultural Center, Parlier, California, in 2012 and 2013

QTL	Scoring time (year, days after planting)	Linkage group	Position (cM)	Flanking markers	LOD	Phenotypic variance explained (%)	Additive effect^a^
*QAc*-*vu1.1*	2012, 60 DAP	1	19	1_0357–1_0312	4.3	10.0	0.97
	2012, 80 DAP	1	19	1_0357–1_0312	5.0	13.3	1.12
	2013, 50 DAP	1	18	1_0357–1_0312	2.4	4.8	0.68
	2013, 70 DAP	1	17	1_1111–1_0357	3.6	7.8	0.75
*QAc*-*vu7.1*	2012, 60 DAP	7	22	1_0912–1_0391	17.6	65.7	2.52
	2012, 80 DAP	7	22	1_0912–1_0391	15.6	61.0	2.43
	2013, 50 DAP	7	22	1_0912–1_0391	16.8	64.2	2.51
	2013, 70 DAP	7	22	1_0912–1_0391	17.1	62.7	2.15

^a^Alleles from susceptible CB27 contribute to higher phenotypic values (damage symptom scores)

### Validation of QTL dominance effect

All highly resistant F2 individuals based on phenotype were either homozygous or heterozygous for IT97K-556-6 alleles at markers flanking the major QTL *QAc*-*vu7.1* (1_0192 and 1_0391), whereas all highly susceptible F2 plants were homozygous for the susceptible CB50 alleles, except for three individuals 50-002, 50-023 and 50-030 that were heterozygous at one or both flanking SNPs (Additional File 3). No clear association was observed in the F2 between phenotypes and genotypes at markers flanking the minor QTL *QAc*-*vu1.1* (1_0357 and 1_0312), and the number of crossovers was high (8 out of 120 F2 genotyped). Source sequences and KASP profiles of SNPs flanking the two QTLs are provided in Additional File 3.

### Physical mapping and legume synteny

Markers flanking the minor QTL *QAc*-*vu1.1* (1_0357 and 1_0312) were positioned in two separate BAC physical contigs (407 and 674), whereas those flanking the major QTL *QAc*-*vu7.1* (1_0192 and 1_0391) were located in the same contig 337 (Additional File 4). BLAST search (Additional File 5) for BAC sequence nodes flanking *QAc*-*vu1.1* (H062E22 and M013N21) identified homologous sequences grouping together in genomes of *M. truncatula* (chromosome 7), soybean (chromosomes 9 and 18), common bean (chromosome 8), pigeon pea (chromosome 3) and mung bean (chromosome 4); genes with strong hits in these regions included those encoding WRKY transcription factors and calcineurin-like and GDSL-like proteins. Likewise, syntenic regions for cowpea BAC sequences flanking *QAc*-*vu7.1* (M016L18, H096J02 and M040H16) were also identified in *M. truncatula* (chromosome 5), soybean (chromosome 1), common bean (chromosome 2), pigeonpea (chromosome 6) and mung bean (chromosome 11); genes with strong hits included those encoding tetratricopeptide, leucine-rich repeats (LRR), nucleotide-binding ARC domain (NB-ARC) and UDP-glucosyltransferase.

## Discussion

To our knowledge, this is the first report on genetic control of aphid resistance in cowpea based on phenotypic data collected from field experiments. Previous studies were mostly performed in greenhouses or screenhouses using artificial inoculation (Githiri et al. 1996; Myers et al. 1996; Pathak 1988; Souleymane et al. 2013) and did not allow identification of linkage groups related to modern consensus maps, precise location or candidate genes. Under field phenotyping, the plants were subjected to natural CPA infestation and other conditions associated with the target environment. However, field design typically is hampered by unpredictable movement of natural CPA and interference by other insect pests. To address this, we planted the highly susceptible cultivar Big Buff along every third row throughout the experimental site. This attracted CPA from adjacent fields, increased population levels and uniformity of infestation, and thereby provided every experimental plot with an equal chance of CPA infestation once they had moved from aphid-damaged Big Buff plants. This enabled successful measurement of resistance reaction among RILs and parents (Fig. 1, Additional File 2).

The continuous bi-modal distribution observed among RILs indicated that CPA resistance was controlled by both major and minor genes. This was confirmed by the genetic mapping of two independent additive QTLs (*QAc*-*vu1.1* and *QAc*-*vu7.1*) using the RIL population (Table 1; Fig. 3). These QTLs may be homologous to resistance genes designated by Pathak (1988), based on quantitative analyses of phenotypic data of different F2 and BC1 populations. They may also be homologous to those QTLs reported by Myers et al. (1996) based on RFLP mapping using an F2 population derived from a different cross between a resistant cultivar IT84S-2246-4 and a susceptible wild cowpea NI 963. However, the resistance in IT84S-2246-4 is showing signs of breakdown in West Africa (Fatokun, pers. comm.), where IT84S-2246-4 and its progenies have shown collapse due to aphid attack at the seedling stage. According to a recent survey by Souleymane et al. (2013), the resistance in IT97K-556-6 also did not seem strong when screened against an African CPA population. On the contrary, in California, this breeding line has been highly resistant, suggesting that biotype differences distinguished by the resistance in IT97K-556-6 occur among CPA populations from different cowpea production regions (Hall et al. 2003). A panel of resistant cowpea genotypes including IT97K-556-6 and IT84S-2246-4 is being screened under uniform test conditions with several CPA colonies collected from different cowpea production areas in West Africa and California, to determine CPA biotype status based on the extent of differential interactions between hosts and CPA populations in this cowpea and aphid system.

A QTL with a major effect on flowering time was mapped on linkage group 8 (LOD score 7, explaining 50 % of the total phenotypic variation), with the early-flowering allele contributed from CB27. This flowering time trait did not affect the response of RILs to aphid infestation. The delayed flowering condition contributed from IT97K-556-6 may have been in response to the longer day-length periods experienced in the California main growing season compared to the typical ‘short day-length’ condition experienced in Sub-Saharan Africa cowpea growing zones.

The QTL *QAc*-*vu7.1* might harbor a major resistance gene from IT97K-556-6 given its strong and stable additive effects across years and scoring times (Table 1). This QTL was also apparently dominant based on our validation test using the CB50 × IT97K-556-6 cross in which F2 resistant plants were either heterozygous or homozygous for IT97K-556-6 alleles at both flanking SNPs 1_0912 and 1_0391 (Additional File 3). Based on physical mapping, SNP markers flanking *QAc*-*vu7.1* were located in the same BAC physical contig of a reference cowpea genome (Additional File 4), providing a confined framework for positional cloning of candidate genes under this QTL. Among candidates with strong BLAST hits to other legume gene models (Additional File 5), UDP-Glycosyltransferases are known to be involved in the biosynthesis of saponins in *Barbarea vulgaris* (Augustin et al. 2012), and these compounds are known to be natural insecticides (De Geyter et al. 2007) which are also present in leguminous plants (Shi et al. 2004). Other candidates included those encoding the NB-ARC domain which is thought to regulate activity of plant resistance proteins (van der Biezen and Jones 1998; van Ooijen et al. 2008). Kamphuis et al. (2012) also reported the co-location of NB-ARC domains and a major QTL for CPA resistance on chromosome 2 of *M. truncatula*. Among candidates found in the minor QTL region (Additional File 5), genes encoding WRKY transcription factors are involved in regulating plant immune responses (Eulgem and Somssich 2007). Together, *QAc*-*vu1.1* and *QAc*-*vu7.1* possibly confer a phloem-based defense mechanism against CPA feeding. Other CPA defense mechanisms might involve variation in aphid attractants such as volatile compounds produced from susceptible plants (Webster et al. 2010). Further investigations on variation in the candidate-gene sequence, expression and biochemical activity among parents and near-isogenic lines with/without IT97K-556-6 alleles at both QTLs could provide insights into pathways of CPA resistance.

## Conclusions

Aphid resistance in cowpea is largely affected by dominant genes based on results from this study and previous research. Due to differential resistance reported for IT97K-556-6 in Africa versus California, the application of the QTLs identified in this study may have more utility against California CPA biotypes like those used in our mapping experiments. Since the delayed flowering trait is independently inherited from aphid resistance, marker-assisted backcrossing could be used to introgress the IT97K-556-6 resistance alleles at the two additive QTLs *QAc*-*vu1.1* and *QAc*-*vu7.1* into susceptible cowpeas without introducing linkage drag for delayed flowering from IT97K-556-6. Using both QTLs may help to promote the durability of resistance by slowing the process of virulence selection and resistance breakdown. Further re-sequencing of the parents and F2/RIL recombinants would be needed for identification of new polymorphisms and breakpoints for subsequent fine mapping of each QTL. Functional analyses of candidate genes based on physical location of the major QTL on linkage group 7 would help identify key gene(s) controlling resistance to California cowpea aphids. In parallel, molecular investigation of California CPA in comparison with African CPA biotypes may provide understanding of how CPA may overcome host resistance in West Africa.

## Electronic supplementary material

Below is the link to the electronic supplementary material.

Additional File 1: The rating scale to assess aphid damage symptoms in the field. (PDF 7492 kb)

Additional File 2: Aphid damage symptoms on cowpea in the field at UC-KARE. (PDF 779 kb)

Additional File 3: Validation of QTL dominance effects in an F2 population. (XLSX 3704 kb)

Additional File 4: The cowpea BAC physical contig harboring the major QTL. (PDF 307 kb)

Additional File 5: Synteny between cowpea and related legumes at two QTLs. (XLSX 85 kb)
