# Supplementary material for: Genetic mapping and legume synteny of aphid resistance in African cowpea (Vigna unguiculata L. Walp.) grown in California
Source: Mol Breed. 2015 Jan 21;35(1):36. doi: 10.1007/s11032-015-0254-0 (PMC4300395; doi:10.1007/s11032-015-0254-0)

Aphid damage symptom – rating scale

Score	0	1	2	3	4	5	6	7	8	9	10
Visible crown damage	no	no	no	no	no	yes	yes	yes	yes	yes	yes
Aphid incidence (%)/plant *(applied to >50% of plants/plot)*	0	1-10	11-20	21-30	31-40	41-50	51-60	61-70	71-80	81-90	91-100

(Next pages are examples of plant appearance for different scores on the aphid damage symptom rating scale)

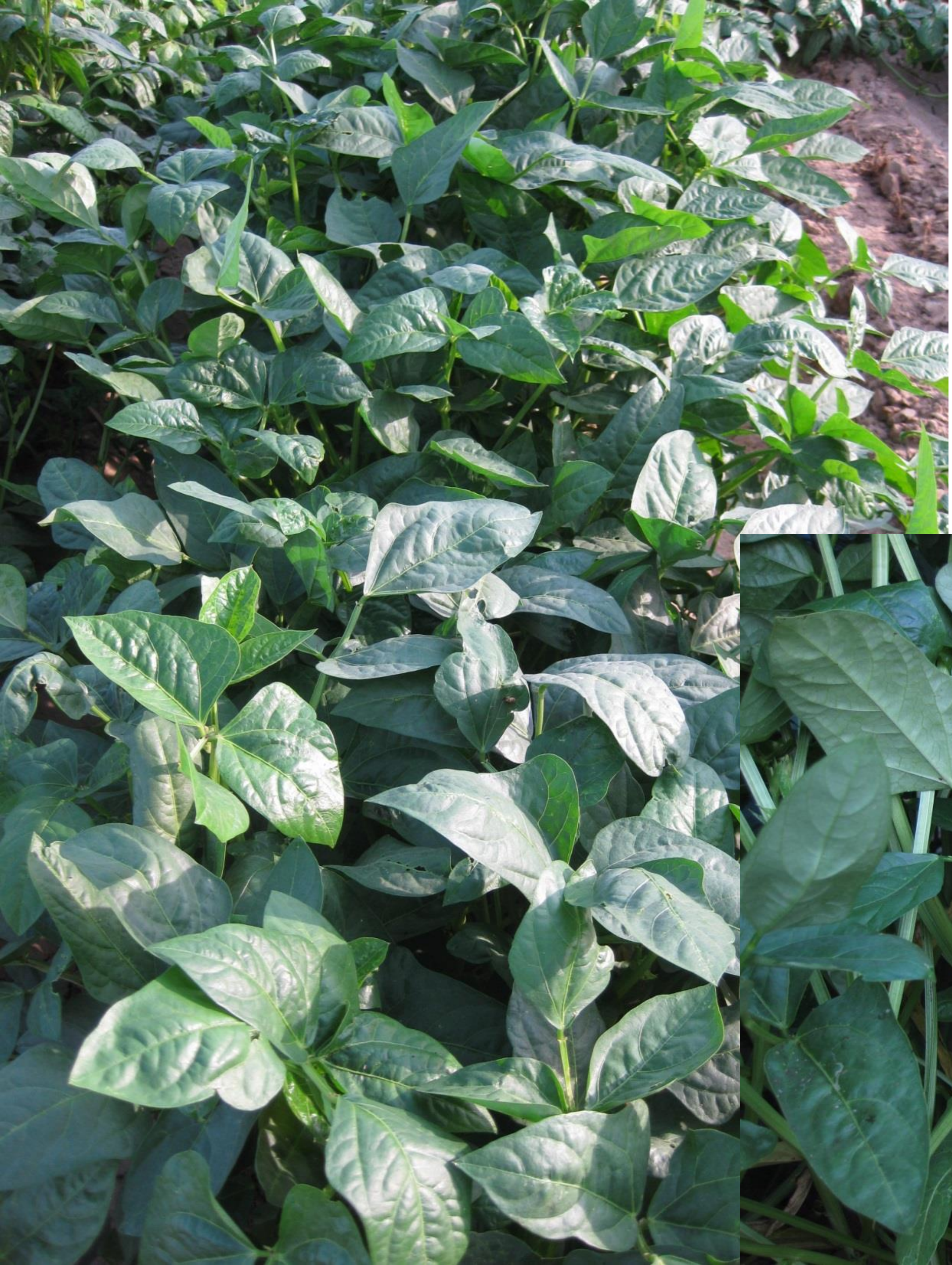

Score 0

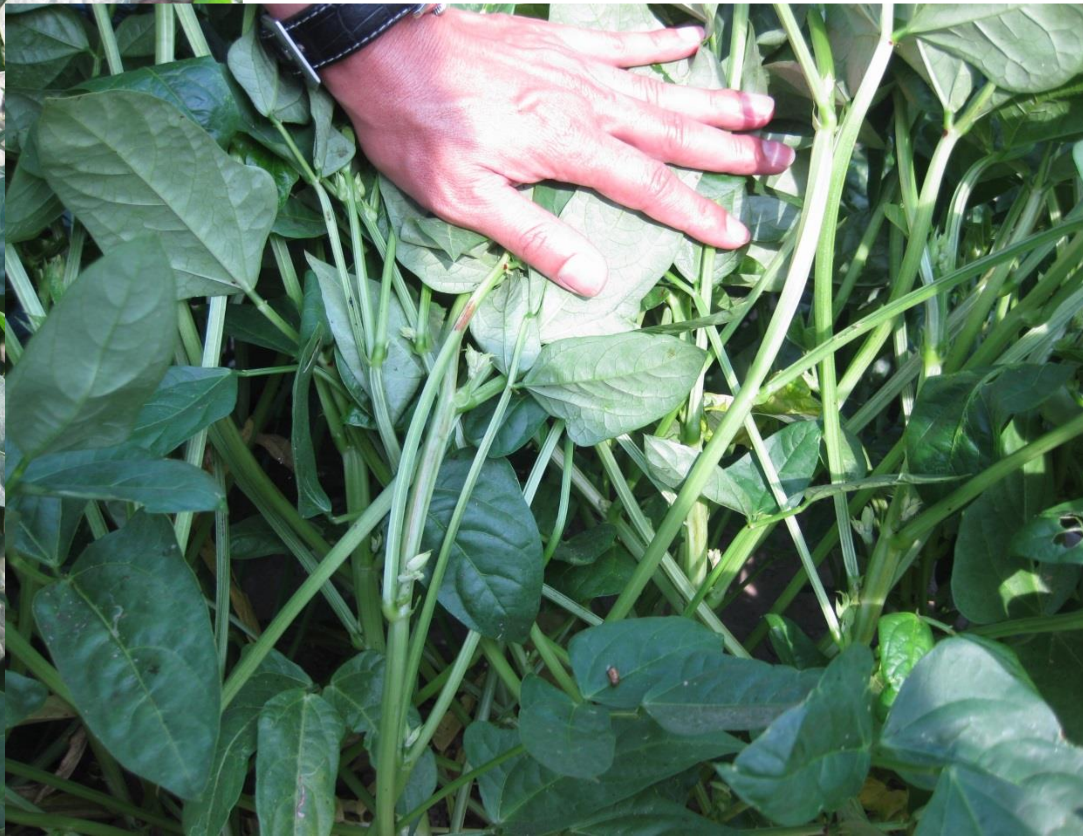

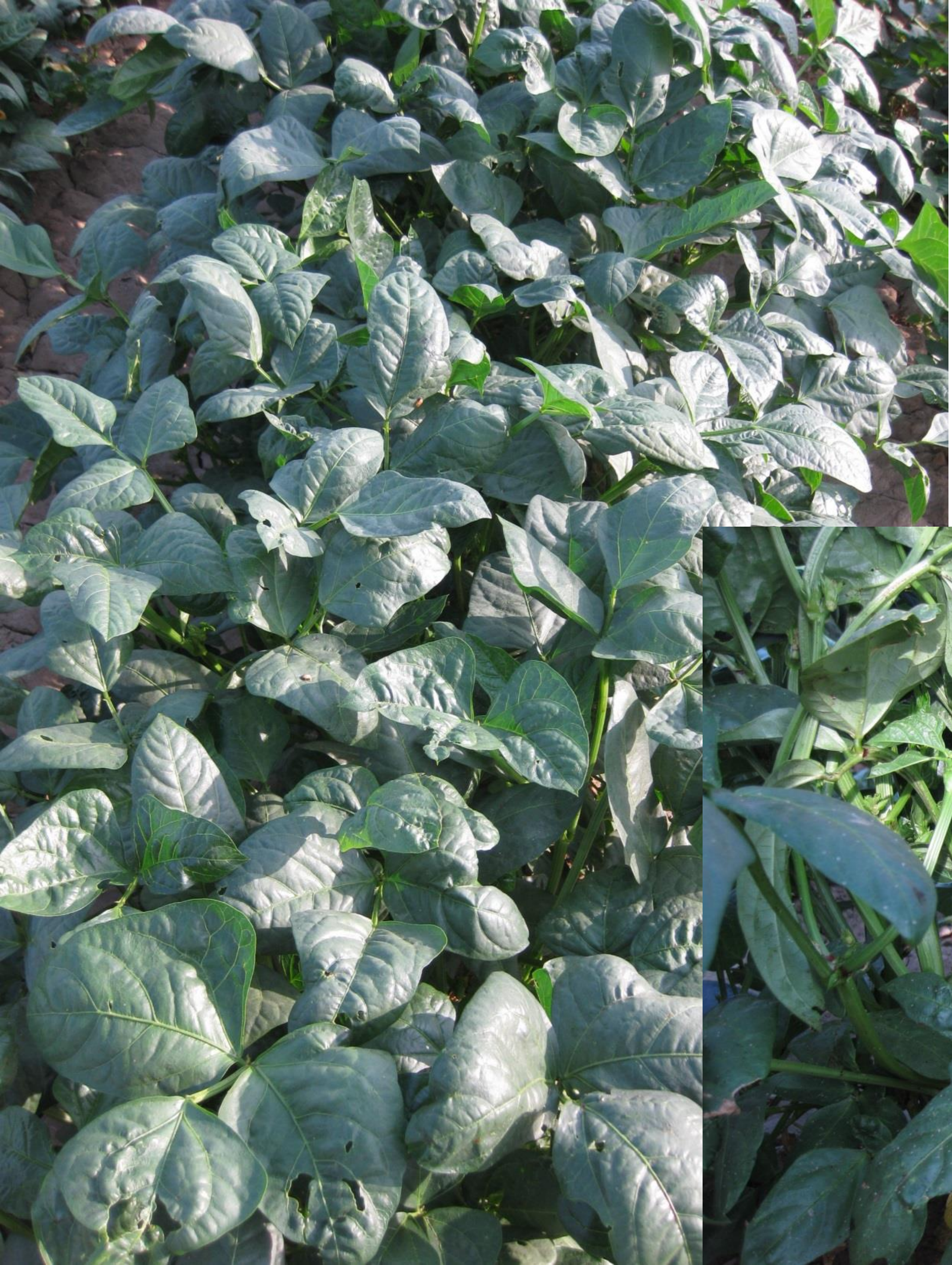

Score 1

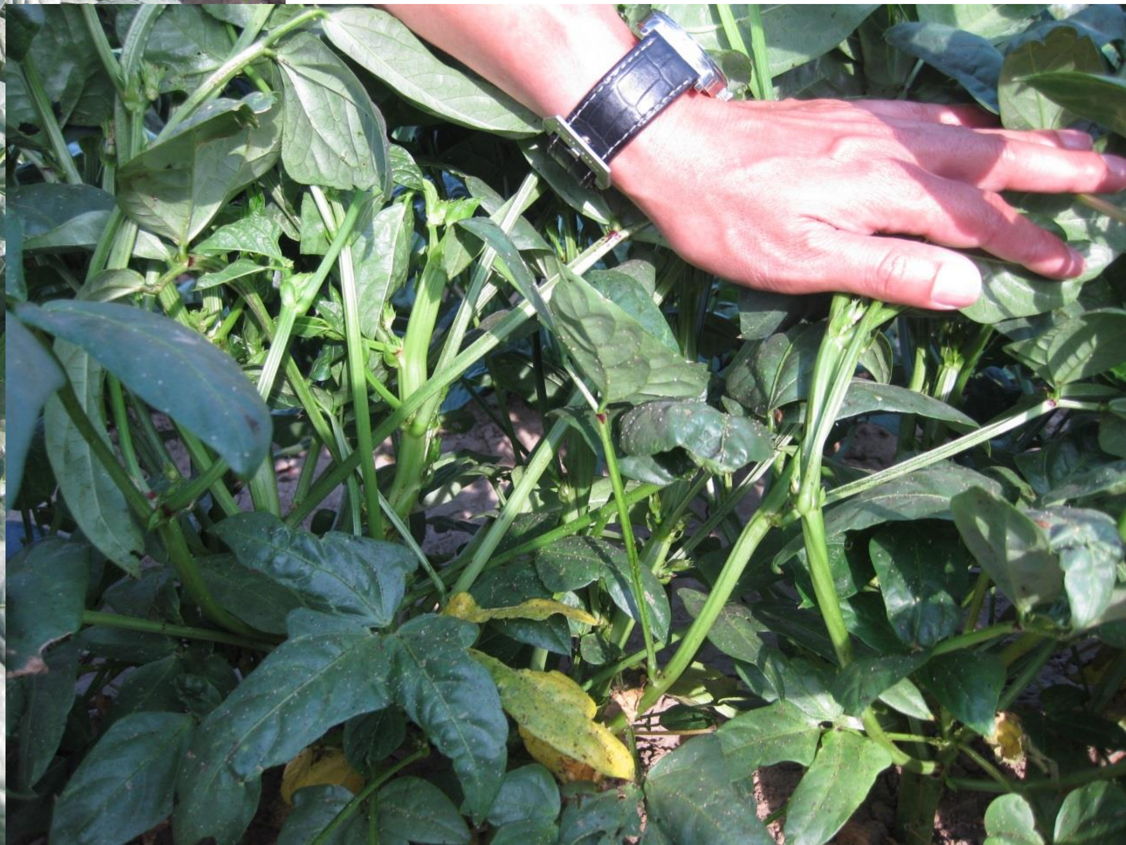

Score 2

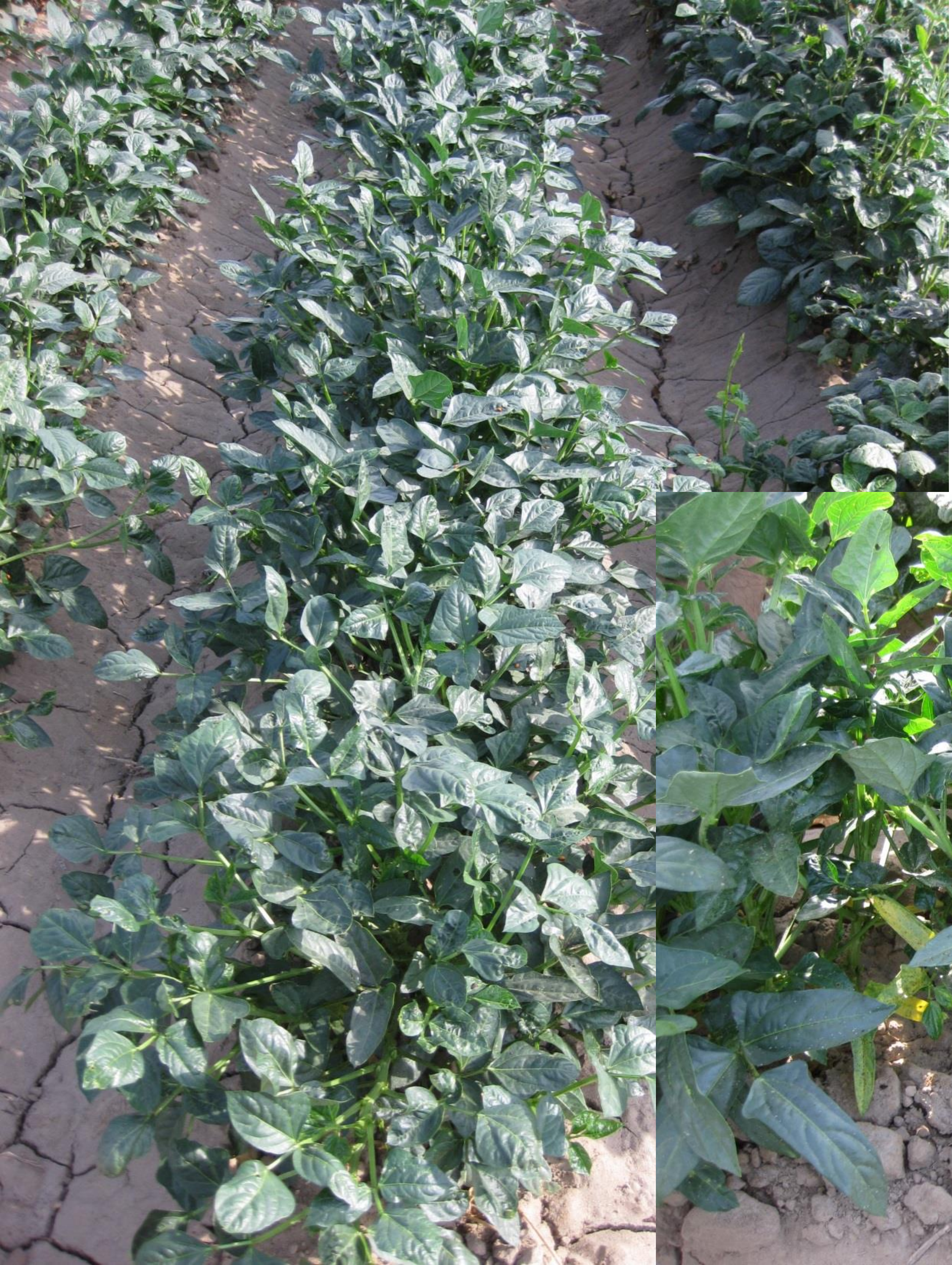

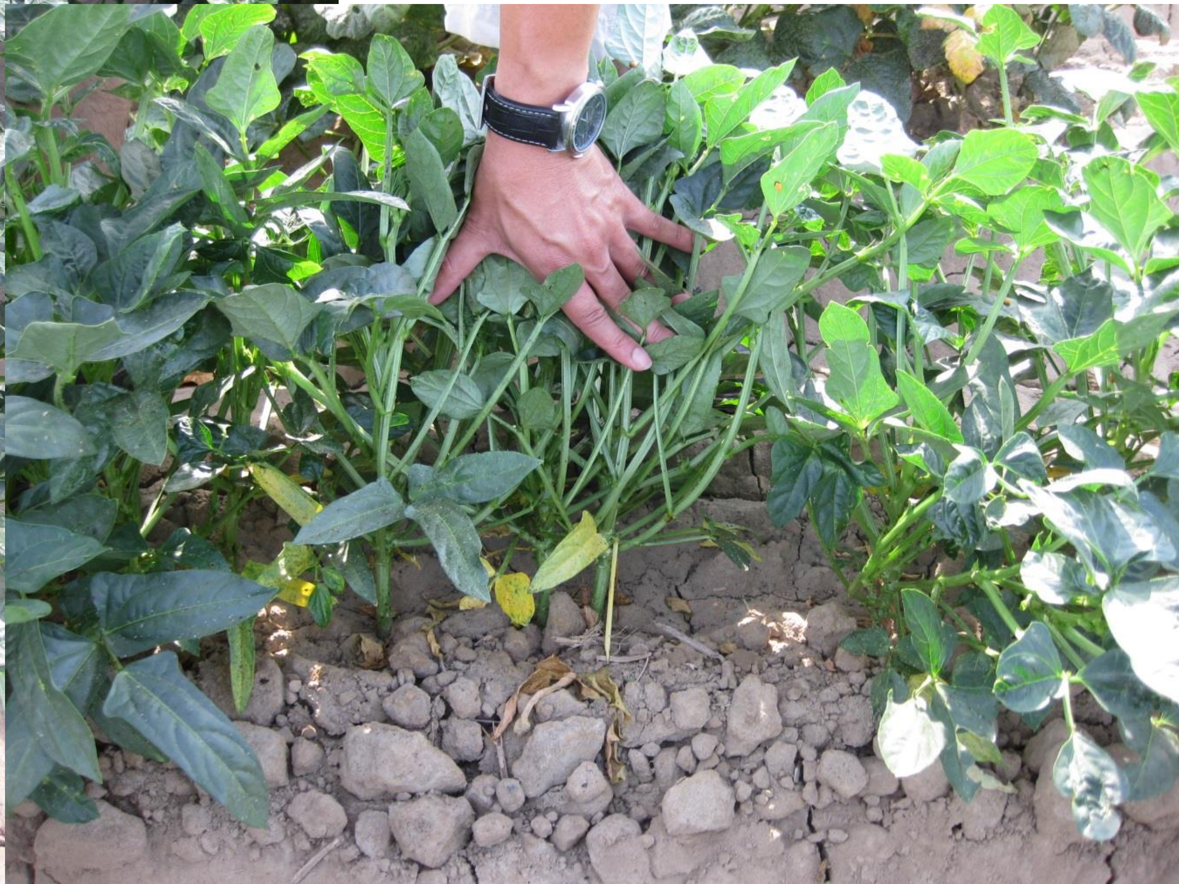

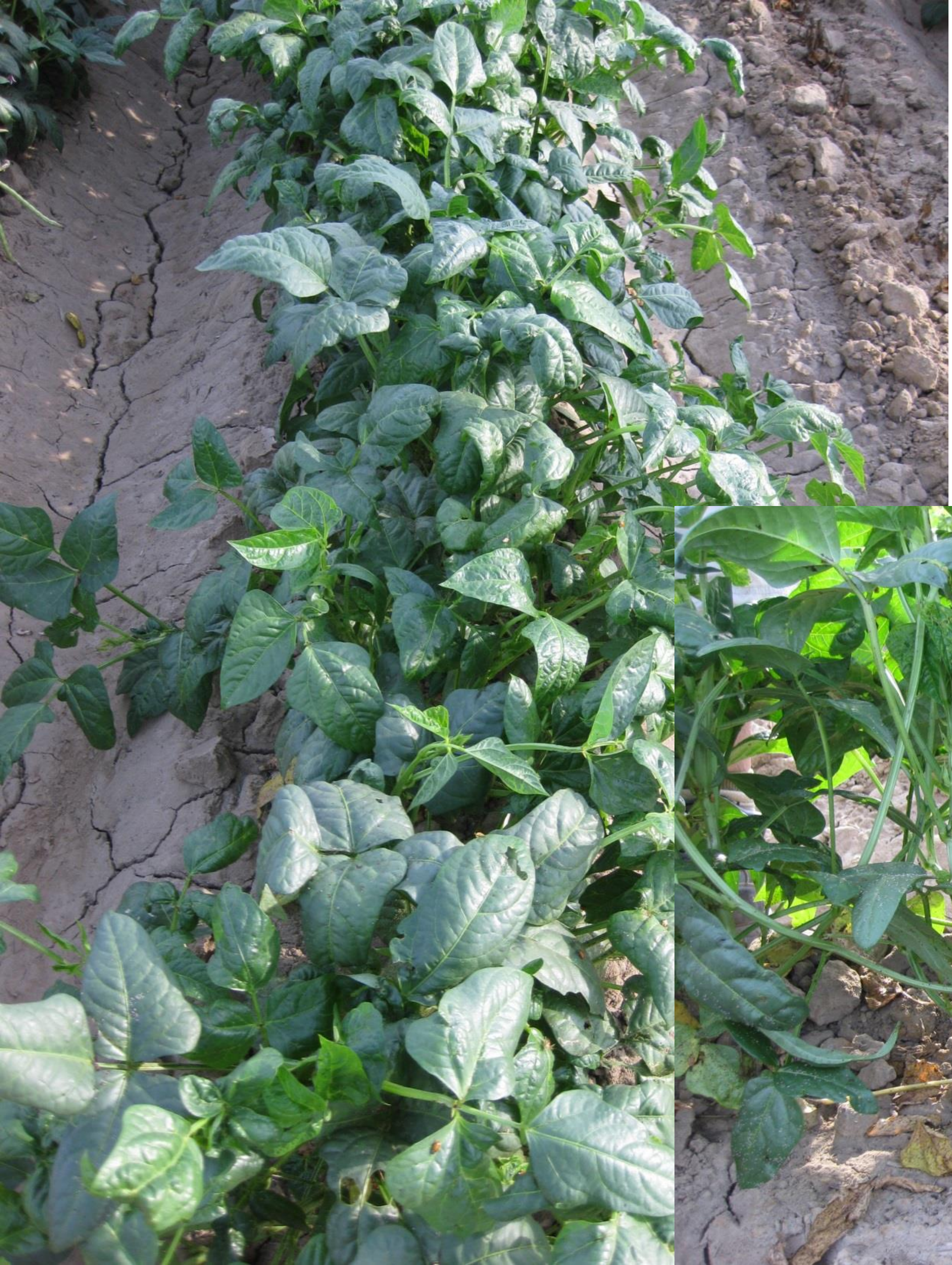

Score 3

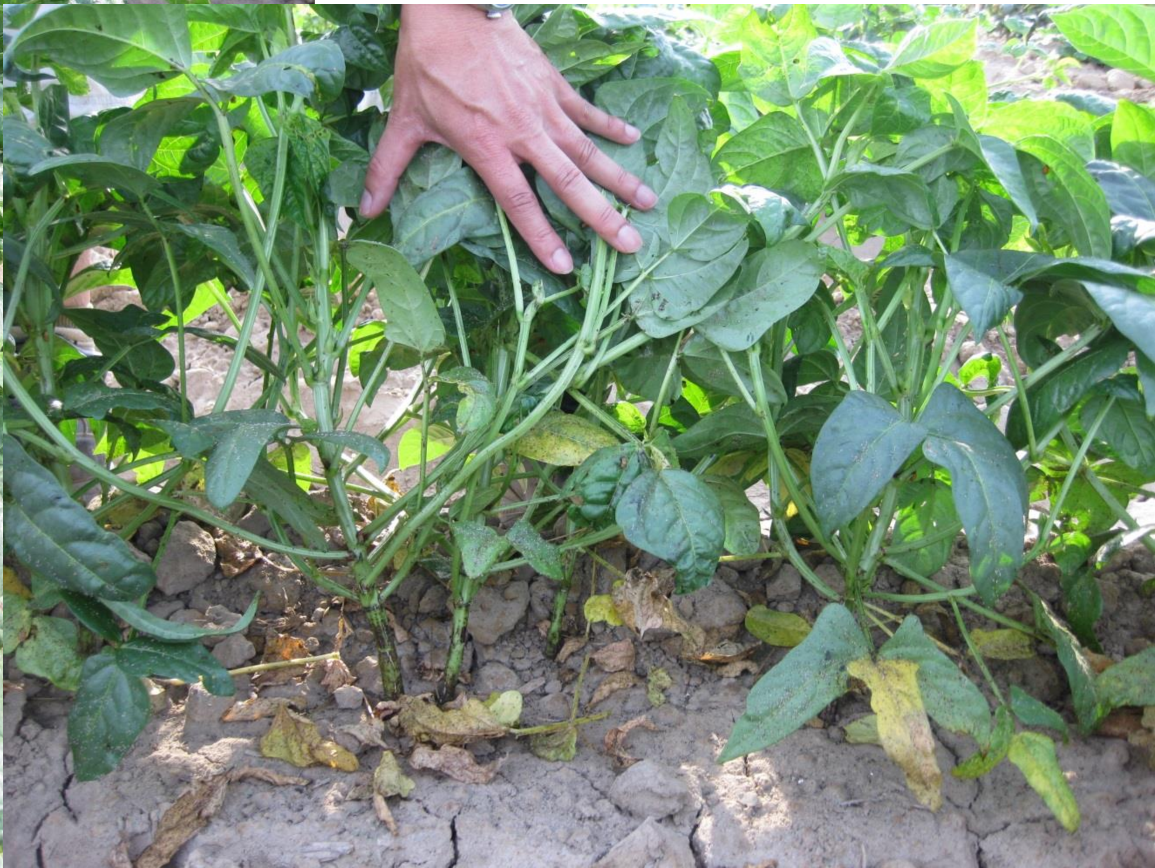

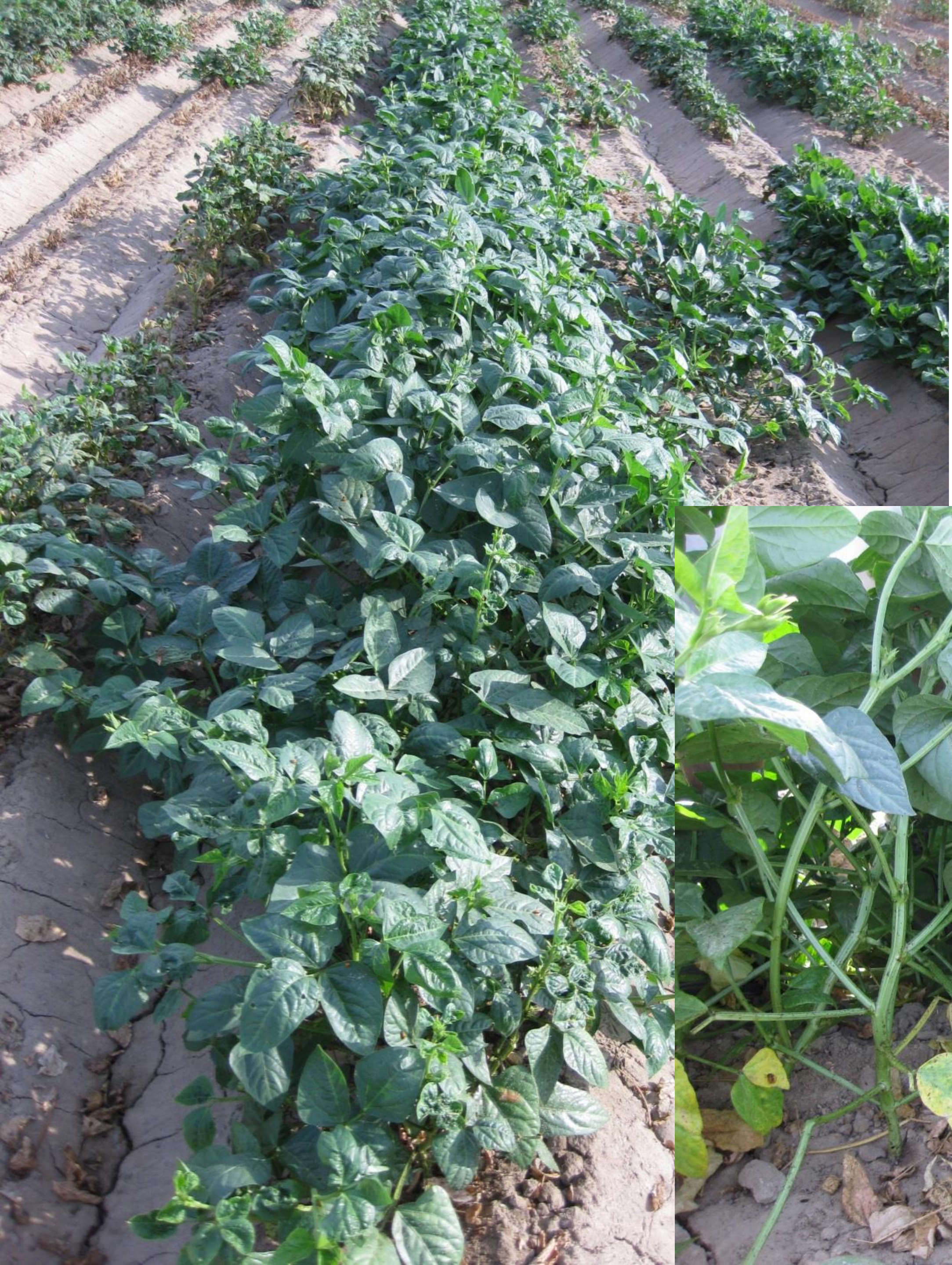

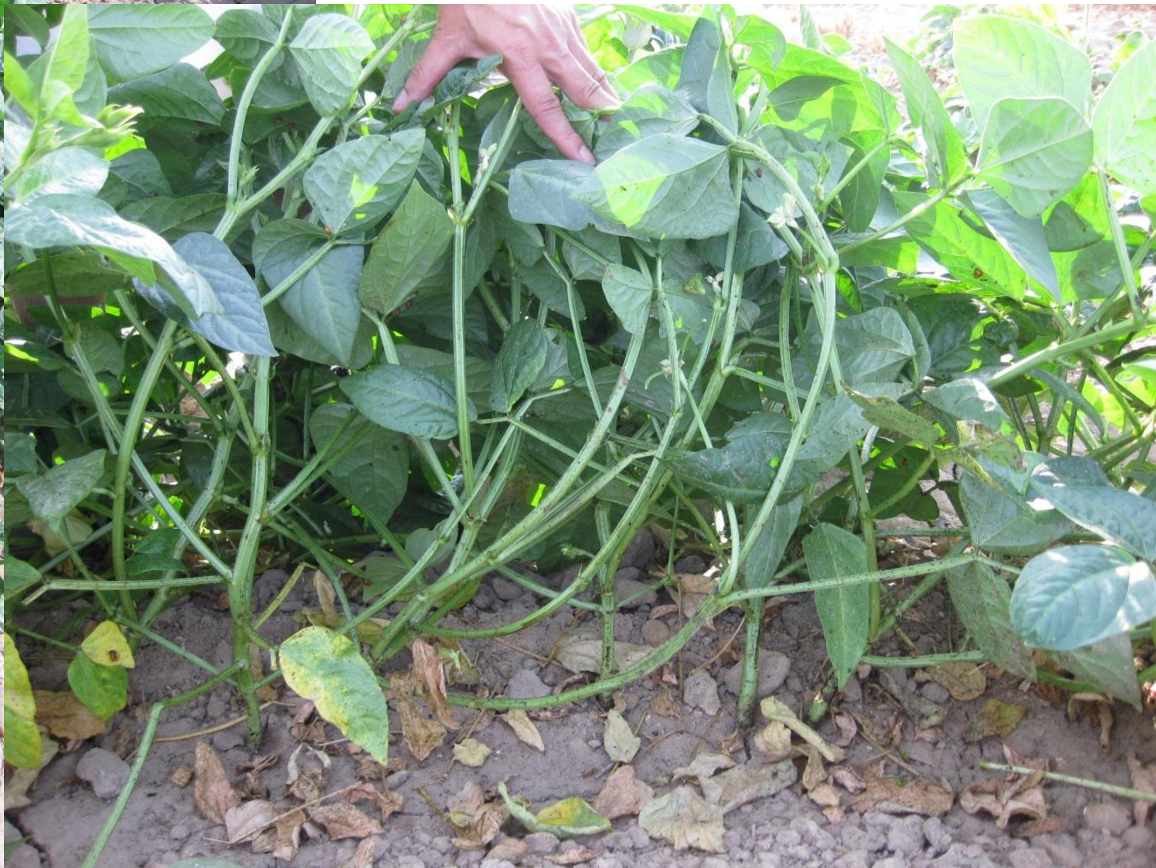

Score 4

Score 5

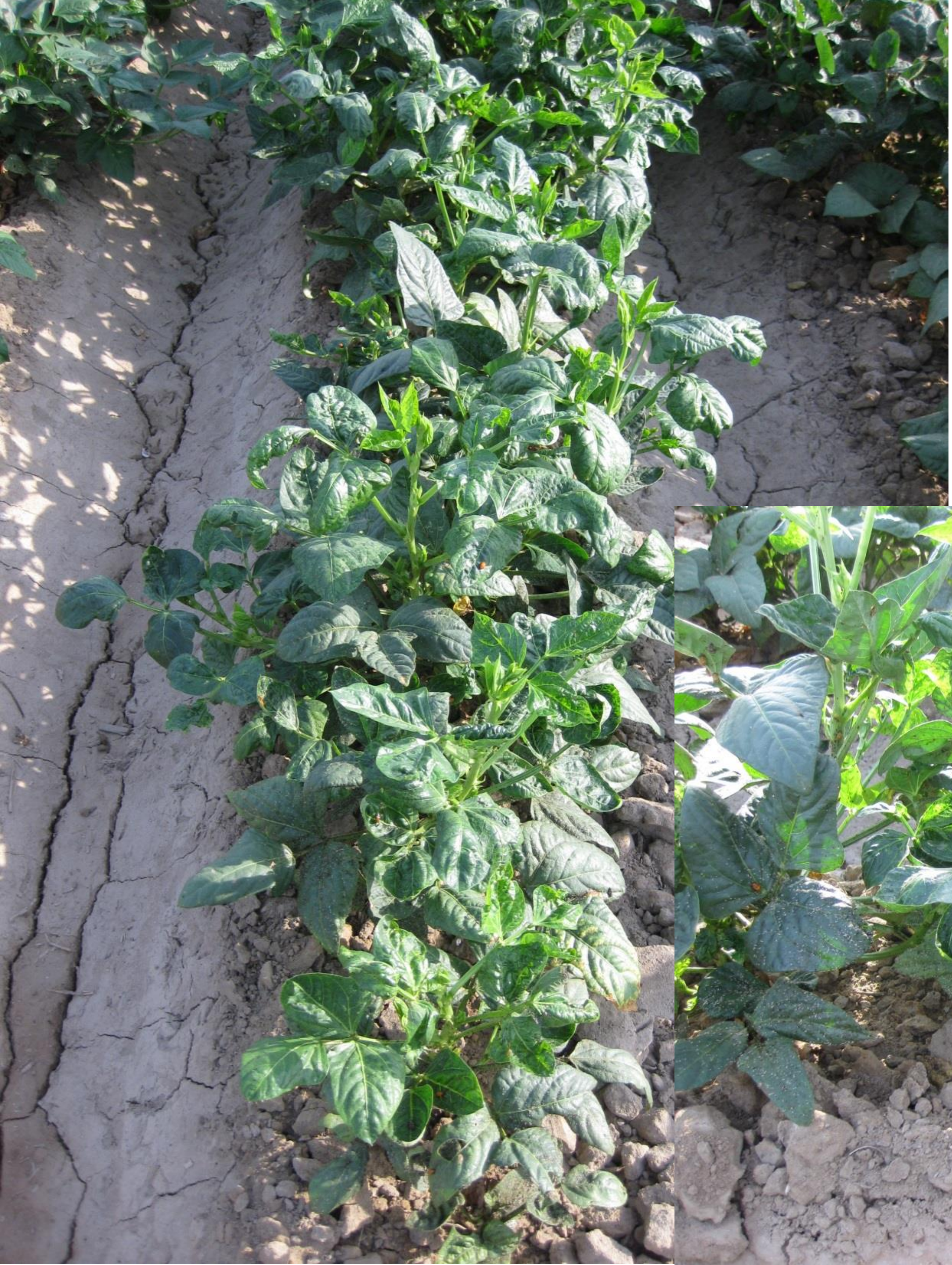

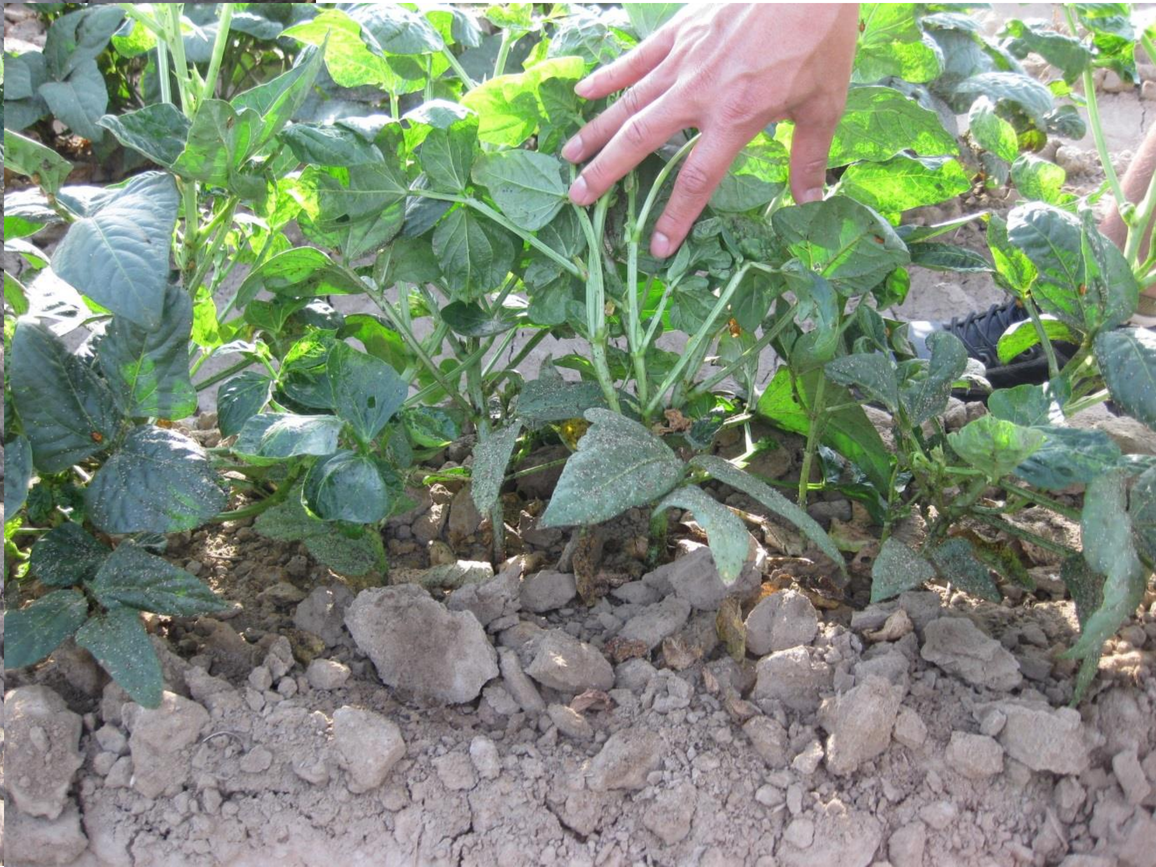

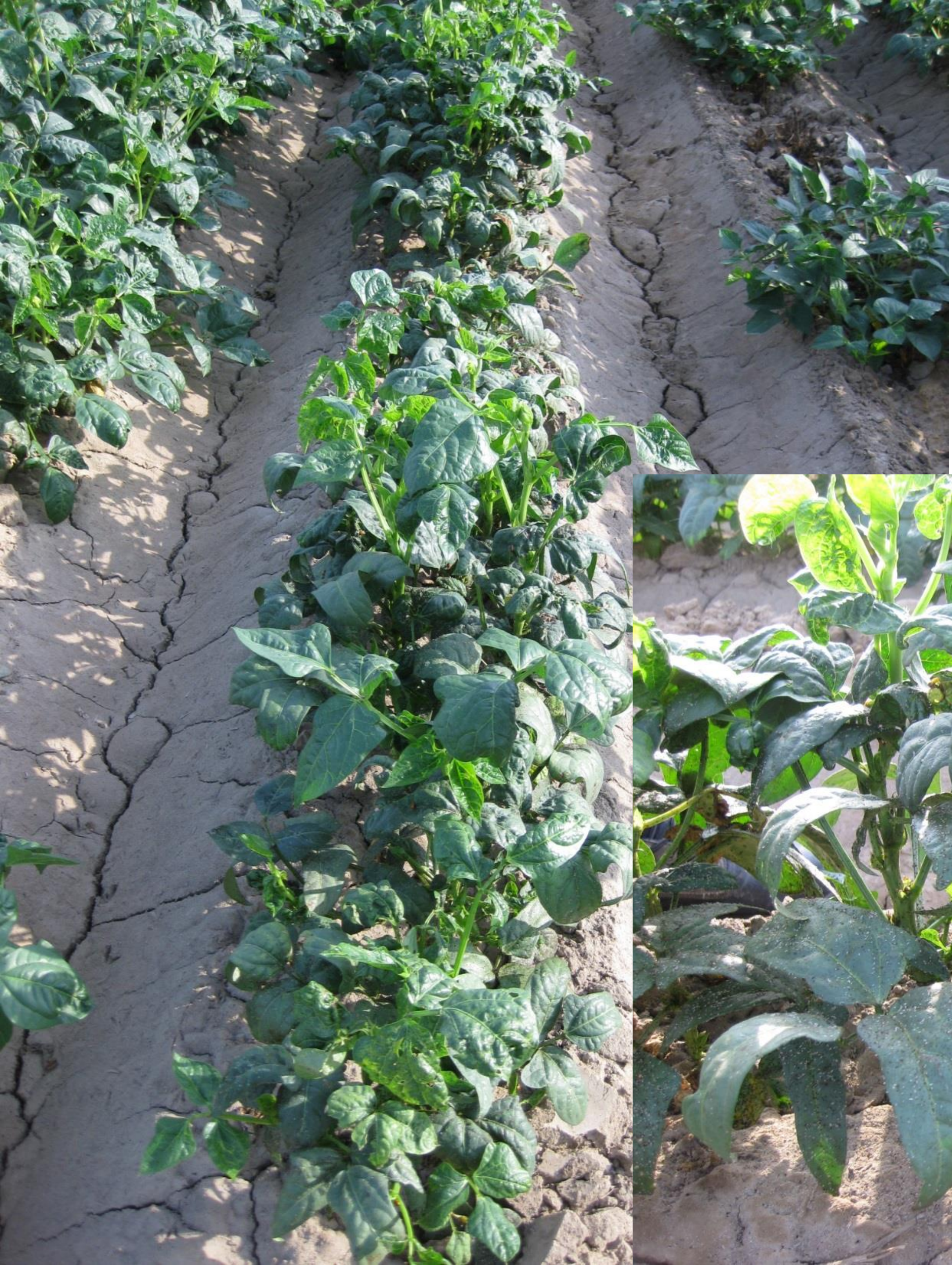

Score 6

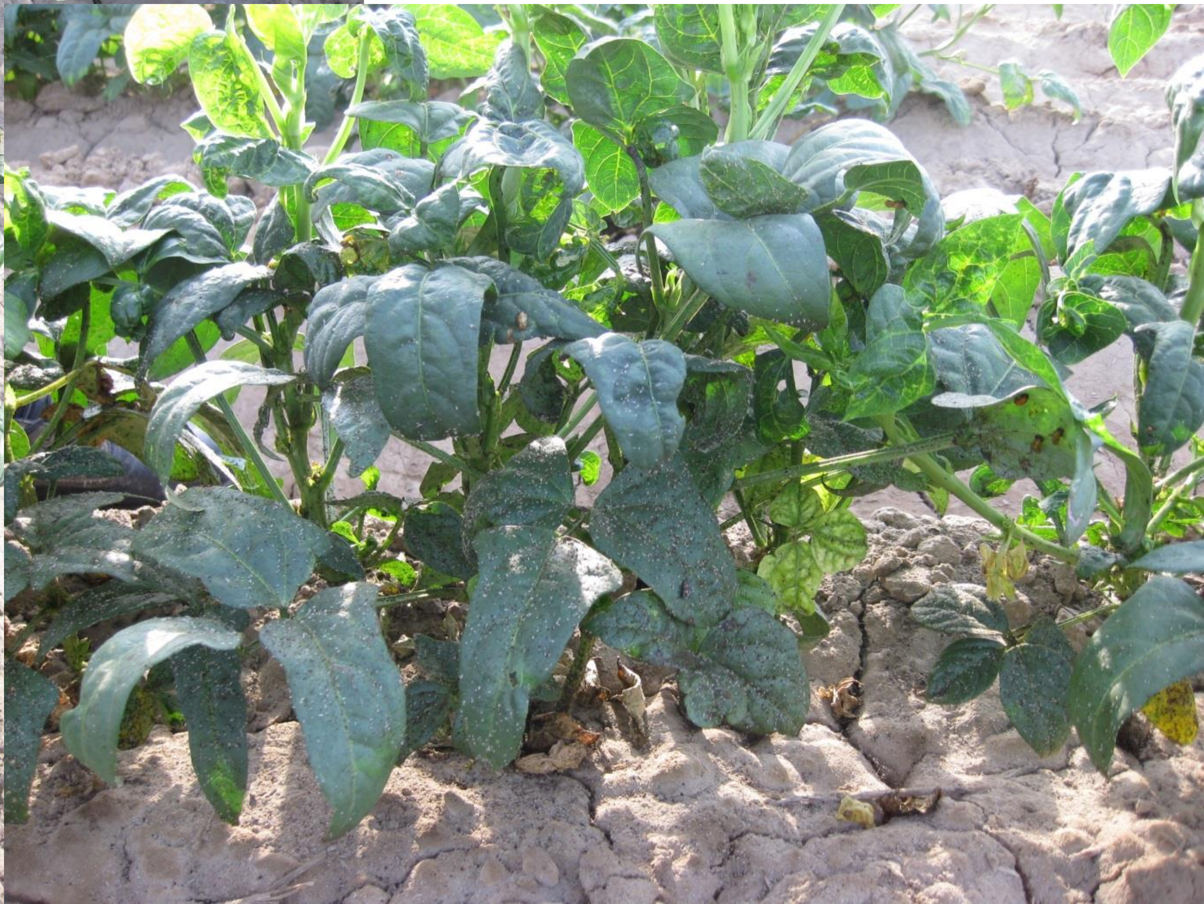

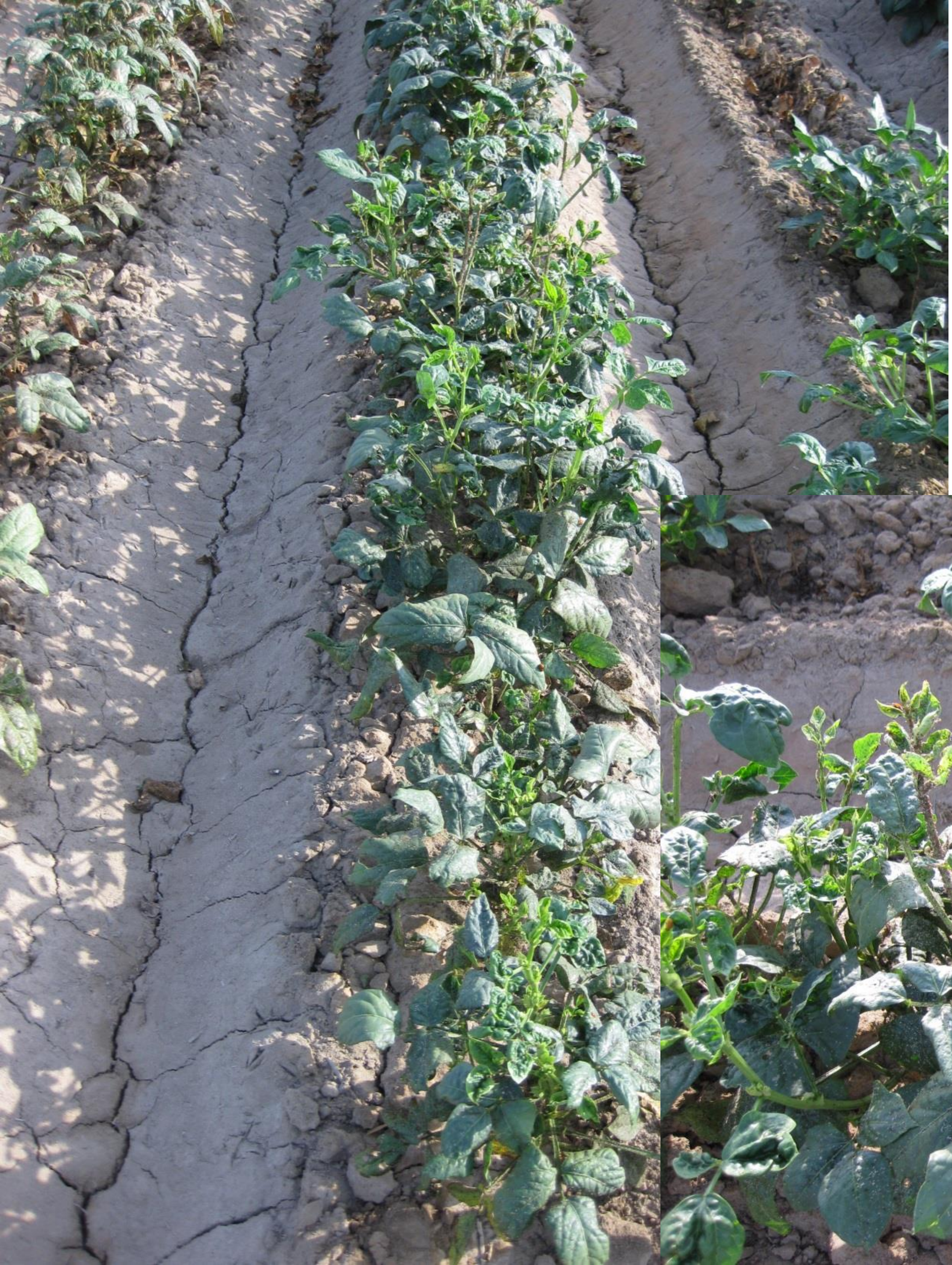

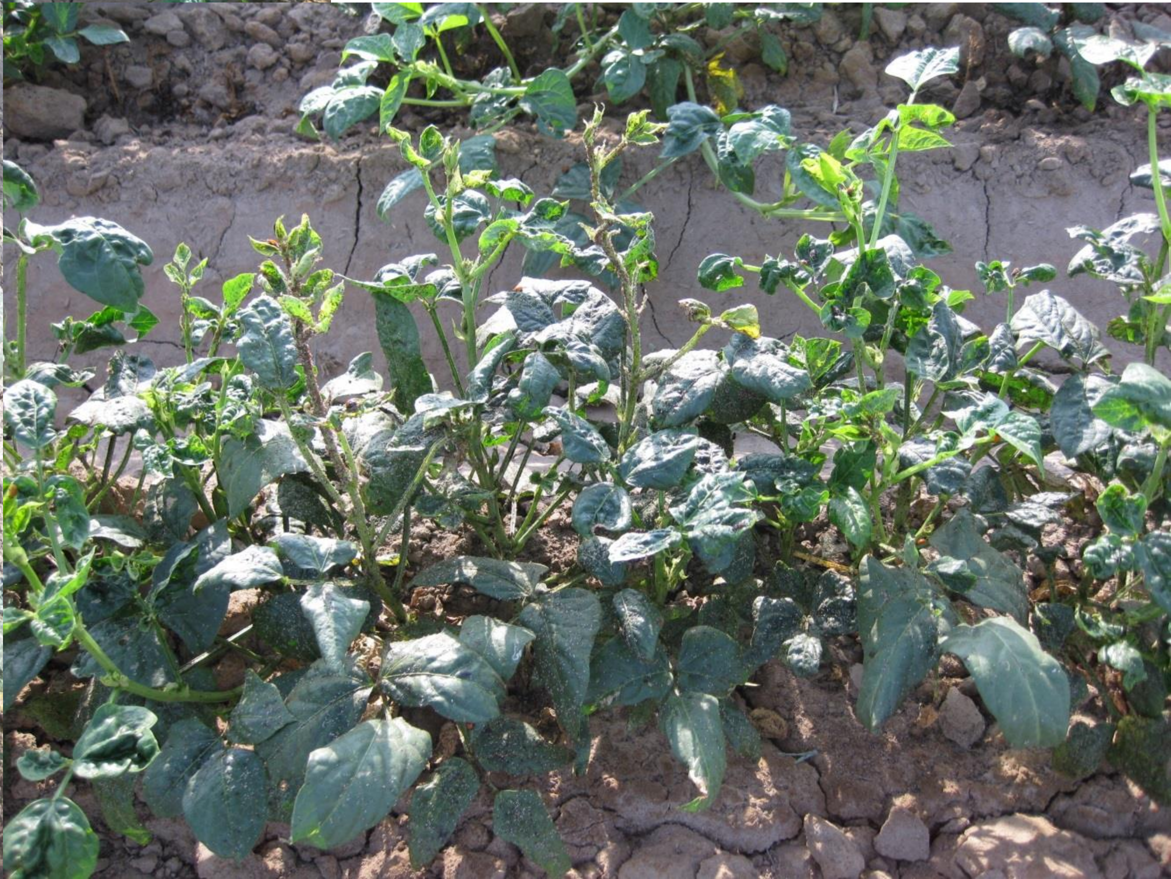

Score 7

Score 8

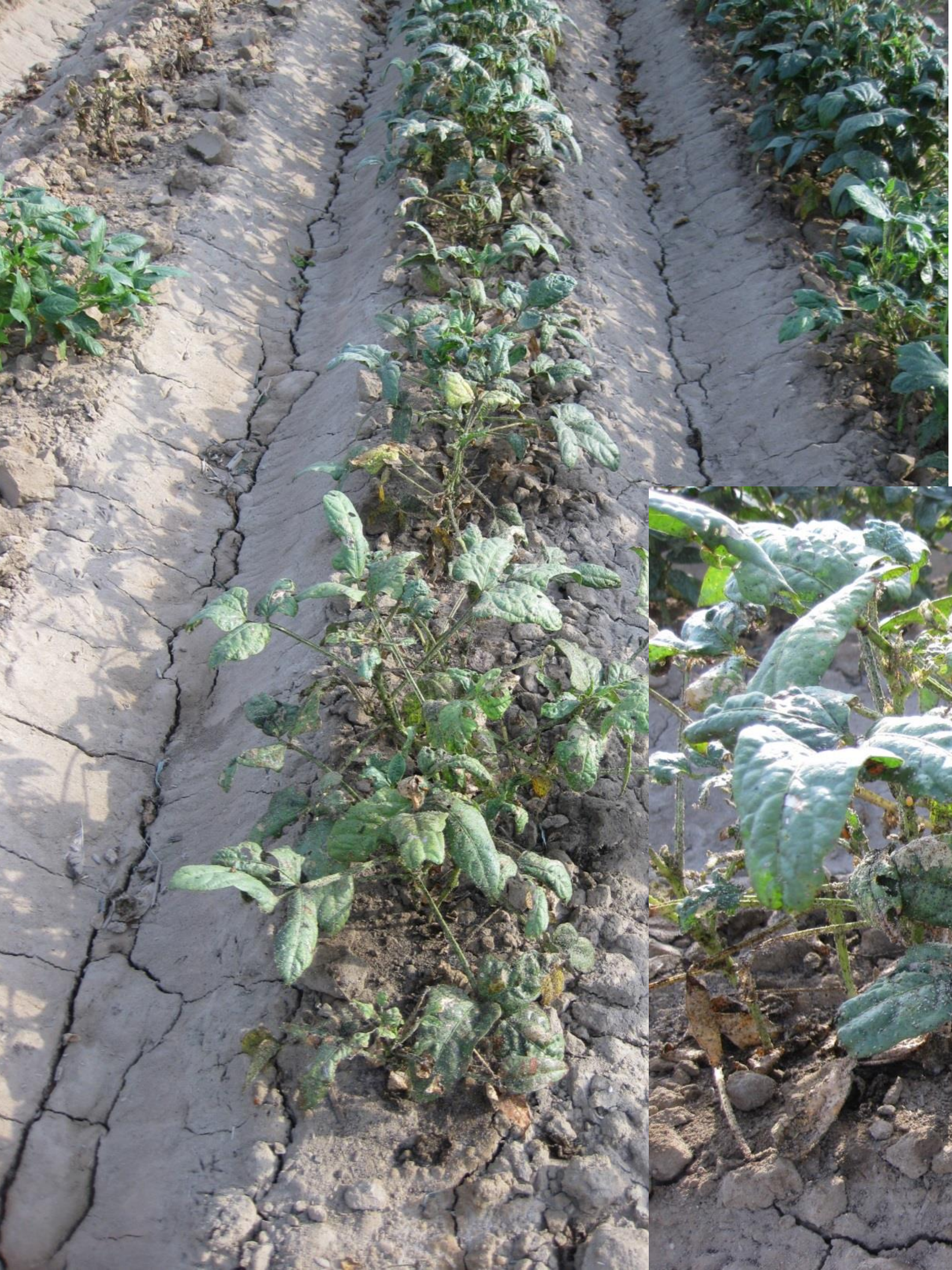

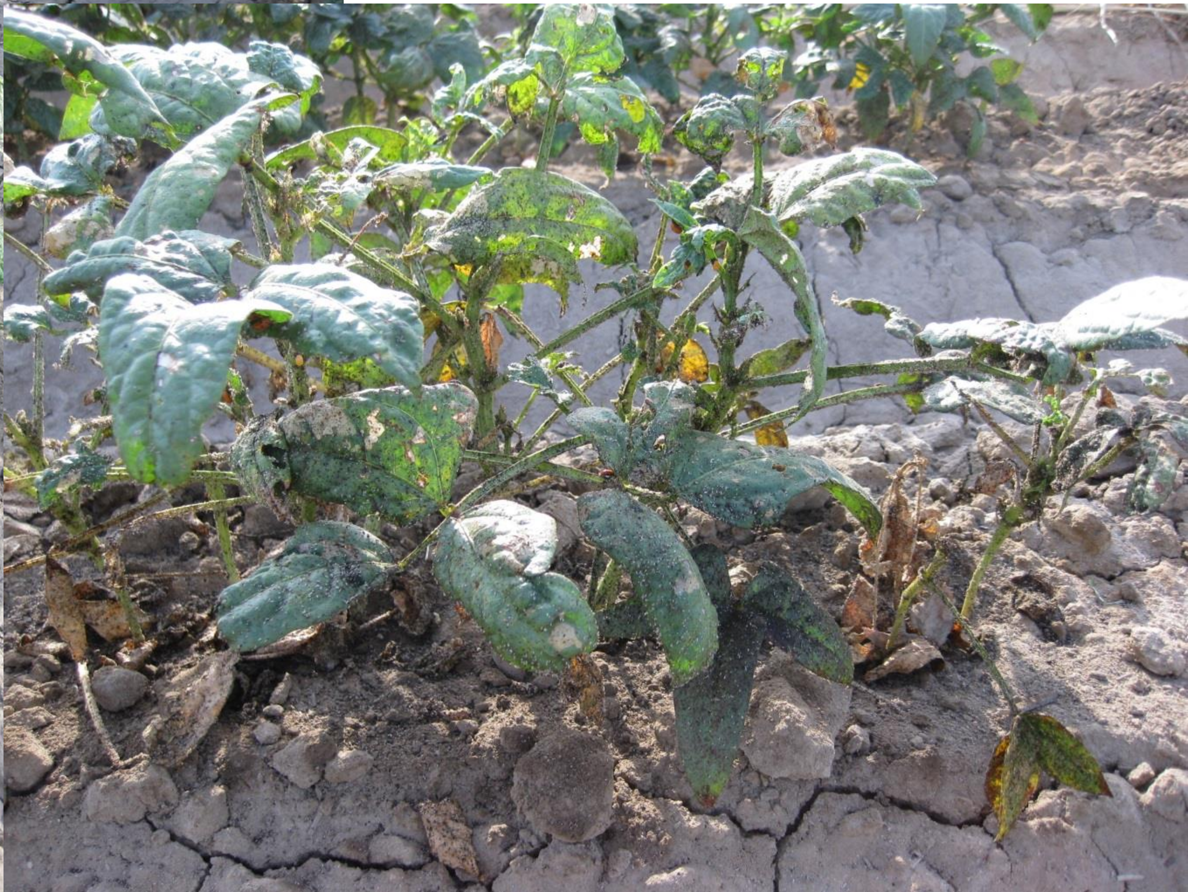

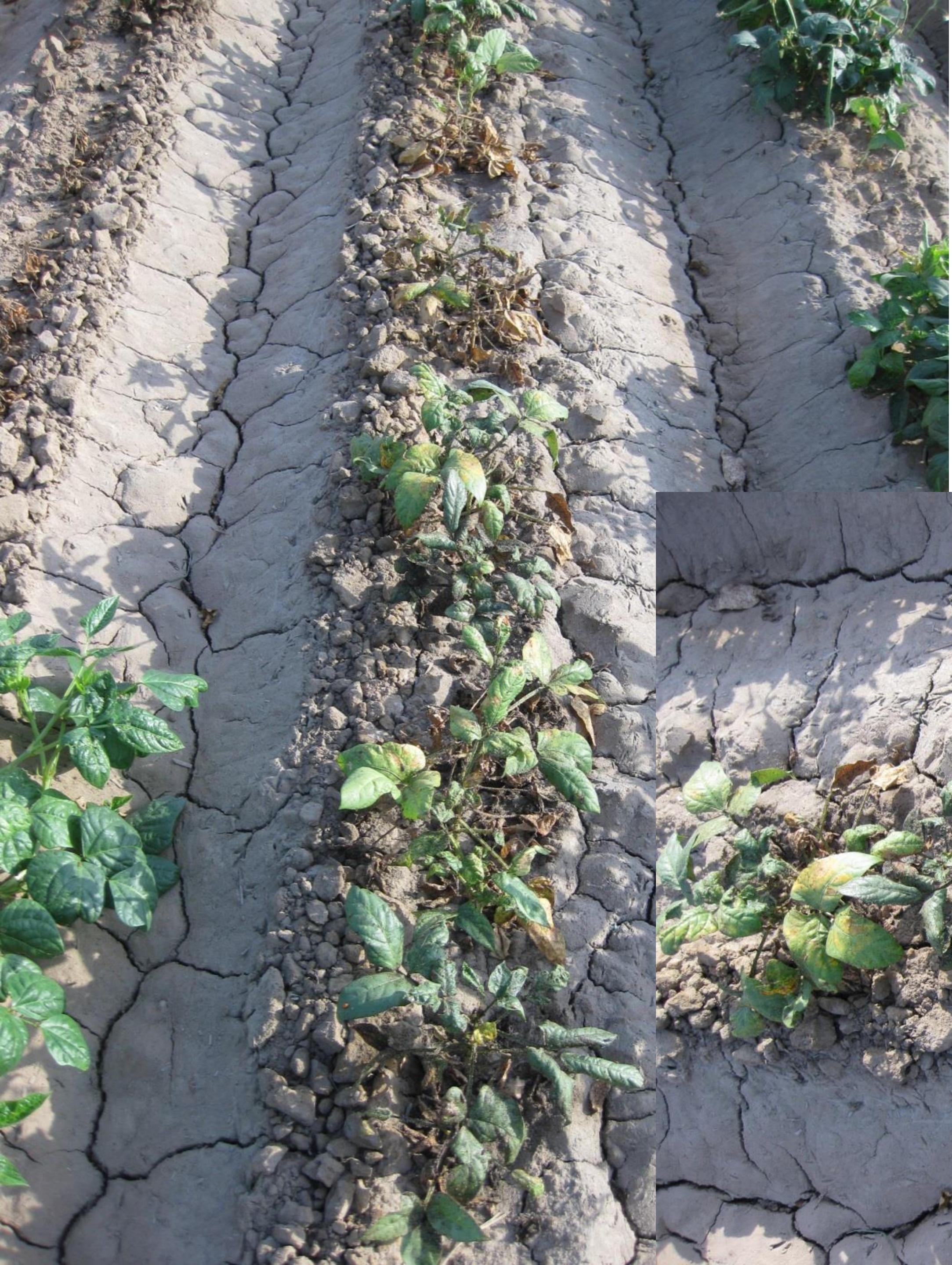

Score 9

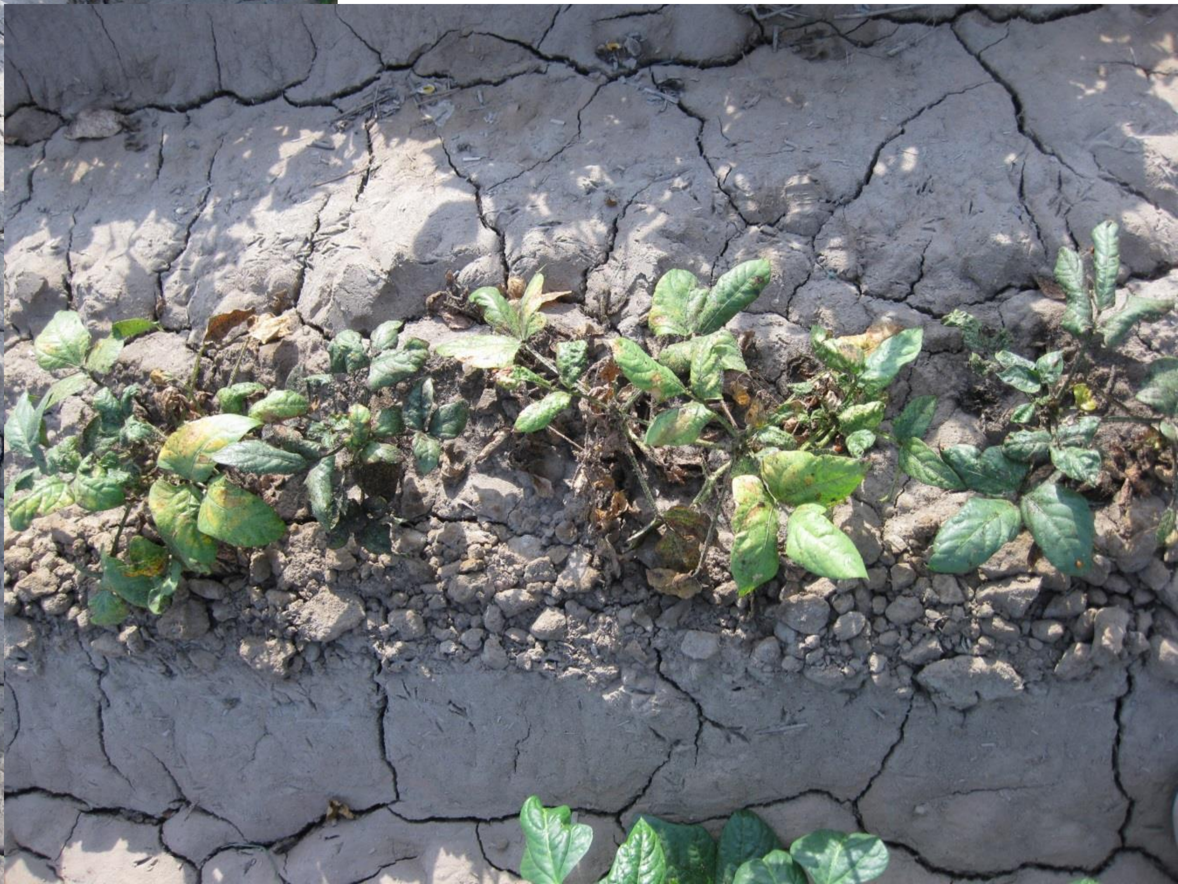

Score 10

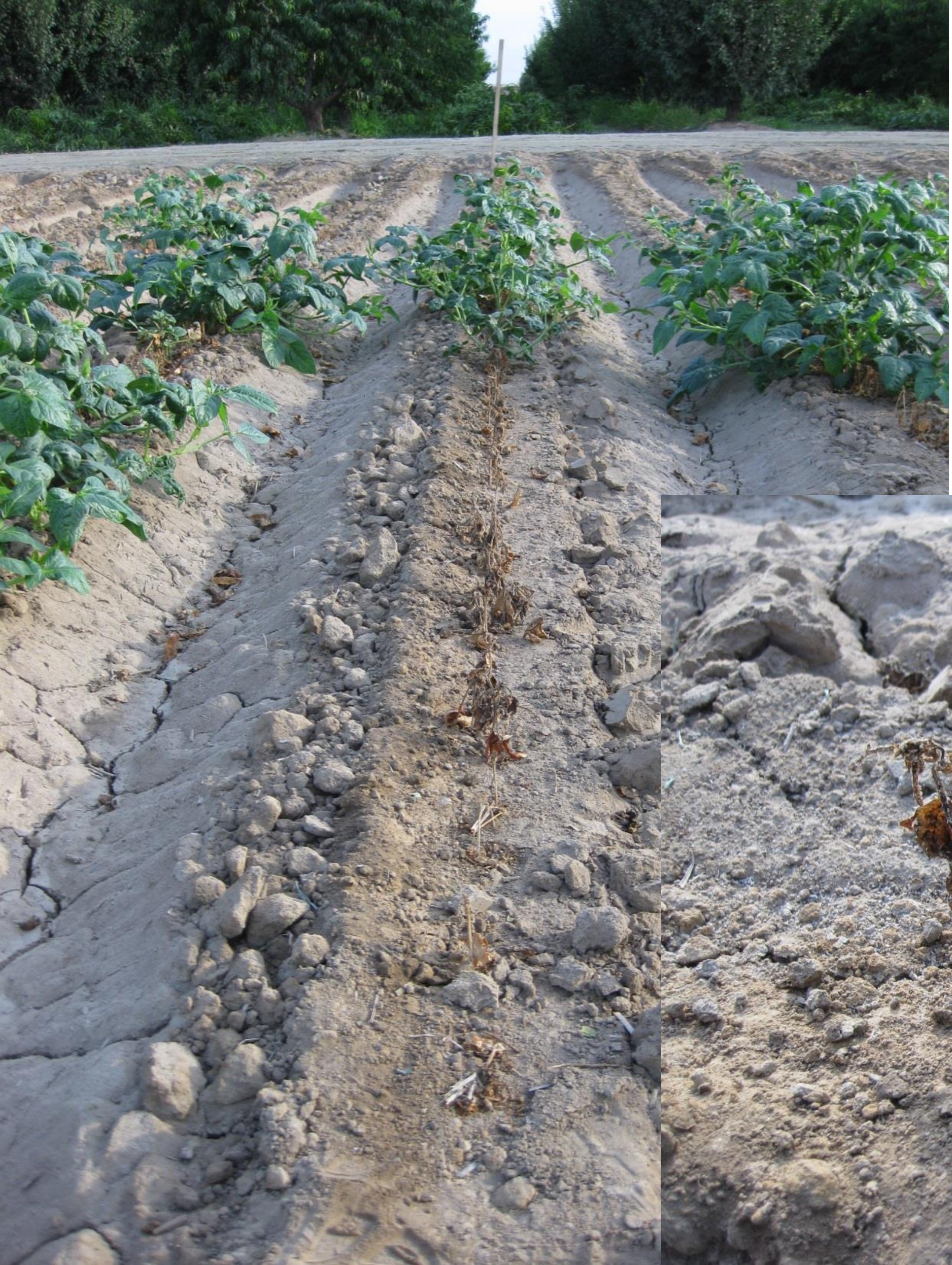

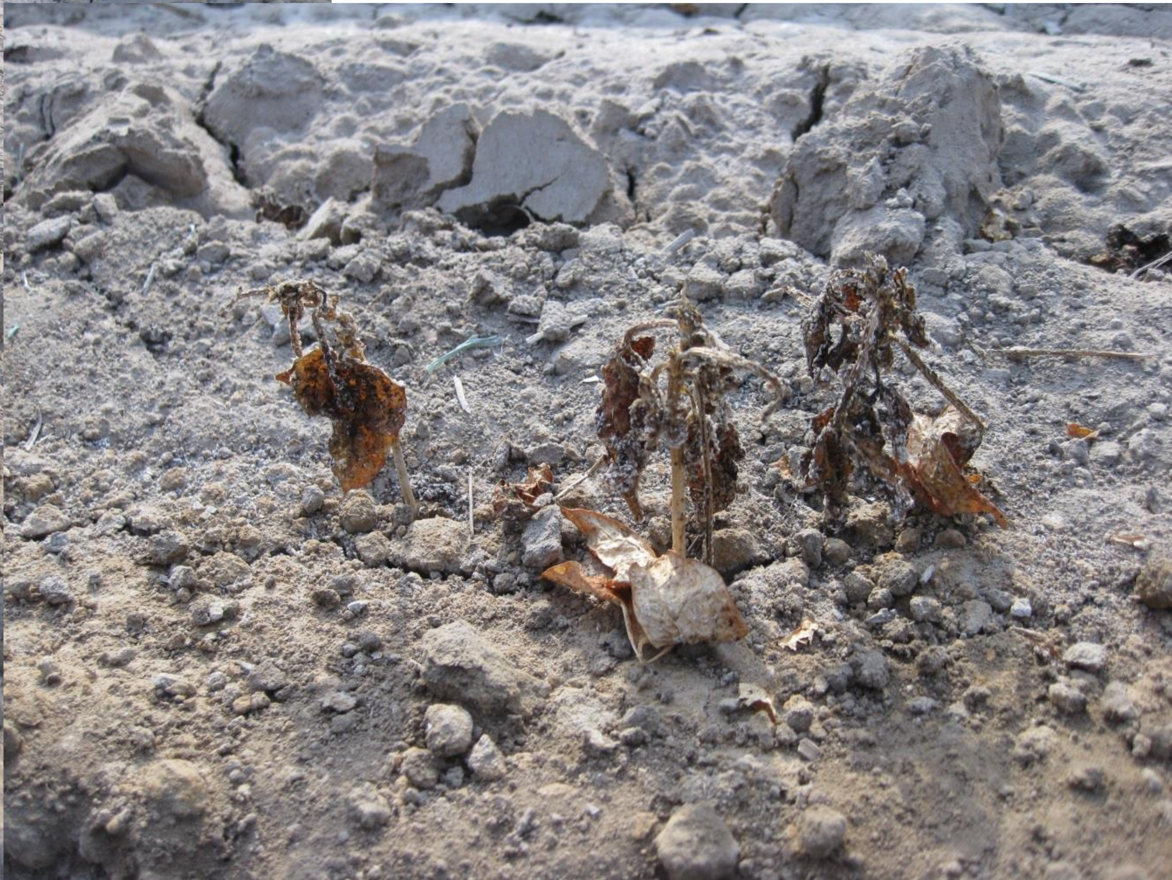

Supplement: Supplementary file 1 — Additional File 1: The rating scale to assess aphid damage symptoms in the field. (PDF 7492 kb) [file 11032_2015_254_MOESM1_ESM.pdf]
