# Supplementary material for: Genetic mapping and legume synteny of aphid resistance in African cowpea (Vigna unguiculata L. Walp.) grown in California
Source: Mol Breed. 2015 Jan 21;35(1):36. doi: 10.1007/s11032-015-0254-0 (PMC4300395; doi:10.1007/s11032-015-0254-0)

Additional File 2. Aphid damage symptoms on cowpea at UC-KARE: (**A**) Aphids feeding on young seedlings: (**B**) nymph and adult aphid microscopic appearance: severe symptoms included (**C**) dead or (**D**) stunted plant with black-mold development caused by aphid honeydew, and (**E**) phenotypic difference in response to aphid infestation between two mapping parents susceptible CB27 (left) and resistant IT97K-556-6 (right).

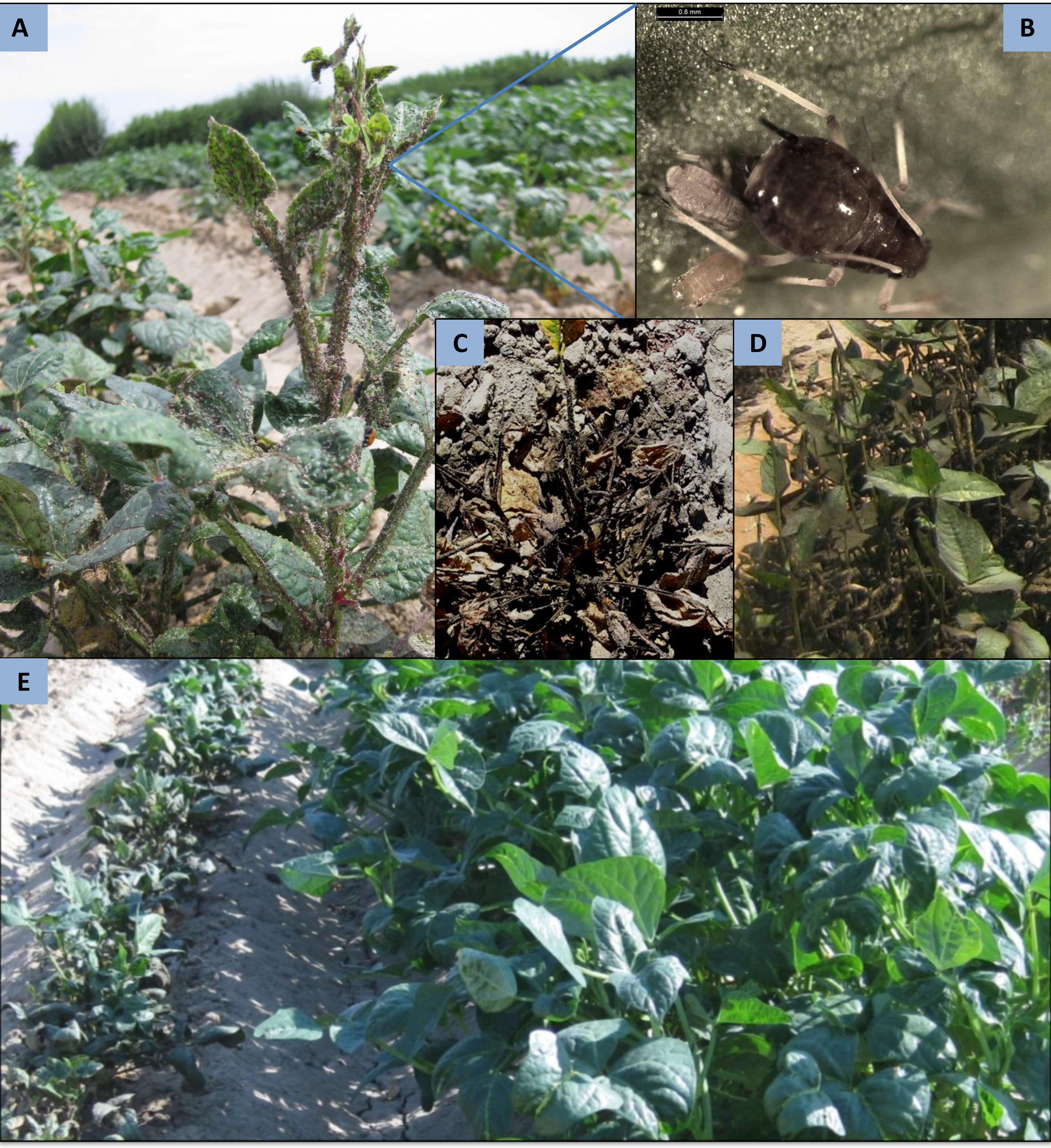

Supplement: Supplementary file 2 — Additional File 2: Aphid damage symptoms on cowpea in the field at UC-KARE. (PDF 779 kb) [file 11032_2015_254_MOESM2_ESM.pdf]
