# Supplementary figures and images for: Genetic mapping and legume synteny of aphid resistance in African cowpea (Vigna unguiculata L. Walp.) grown in California
Source: Mol Breed. 2015 Jan 21;35(1):36. doi: 10.1007/s11032-015-0254-0 (PMC4300395; doi:10.1007/s11032-015-0254-0)

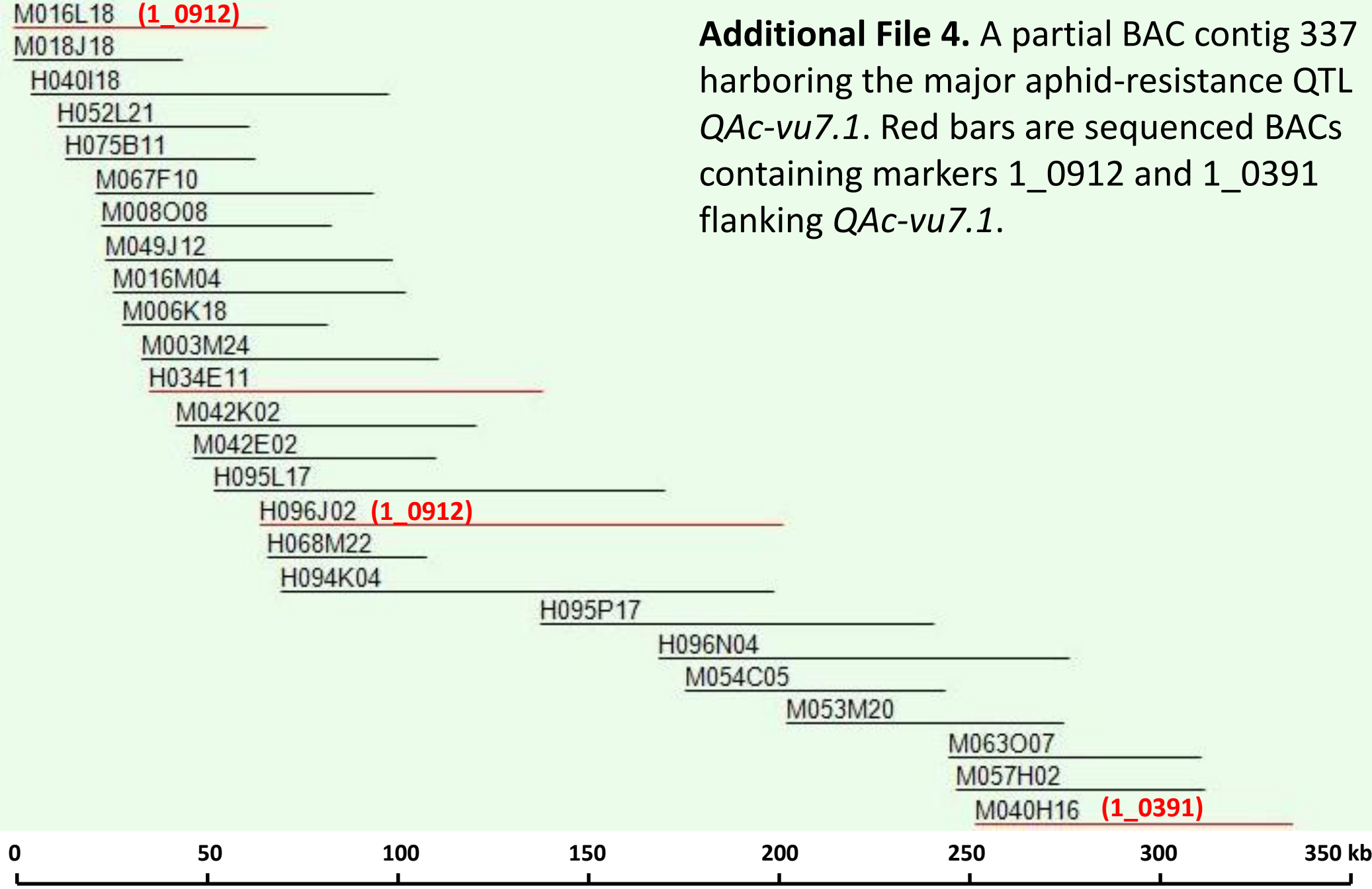

Supplement: Supplementary file 4 — Additional File 4: The cowpea BAC physical contig harboring the major QTL. (PDF 307 kb) [file 11032_2015_254_MOESM4_ESM.pdf]
